# Associations between health-related social capital and oral frailty risk among older adults: A stratified analysis by socioeconomic status in the JAGES longitudinal study

**DOI:** 10.1371/journal.pone.0353850

**Published:** 2026-07-24

**Authors:** Nanako Tomioka, Yuka Iwata, Yuko Tanaka, Ayuka Yokoyama, Kisaki Kobayashi, Akemi Matsuzawa, Toshiyuki Ojima, Etsuko Tadaka

**Affiliations:** 1 Department of Community and Public Health Nursing, Graduate School of Health Sciences, Hokkaido University, Sapporo, Hokkaido, Japan; 2 Department of Community and Public Health Nursing, Faculty of Medicine/Graduate School of Health Sciences, Hokkaido University, Sapporo, Hokkaido, Japan; 3 Department of Pediatric Nursing, Faculty of Medicine/Graduate School of Health Sciences, Hokkaido University, Sapporo, Hokkaido, Japan; 4 Department of Community Health and Preventive Medicine, Hamamatsu University School of Medicine, Hamamatsu, Shizuoka, Japan; Tokyo Metropolitan Institute of Geriatrics and Gerontology, JAPAN

## Abstract

**Background:**

Although socioeconomic inequalities in oral health persist despite universal dental care, the role of social determinants remains unclear. Social capital may influence oral frailty, but evidence is limited, particularly regarding individual- and community-level social capital and differences by socioeconomic status (SES). This study examined these associations among community-dwelling older adults.

**Methods:**

We analyzed longitudinal data from the Japan Gerontological Evaluation Study (JAGES) collected at baseline (2019) and follow-up (2022), including 31,378 older adults. Social capital was assessed across three domains: civic participation, social cohesion, and reciprocity at individual and community levels. Oral frailty was estimated using a validated predictive model incorporating age, remaining teeth, chewing ability, and swallowing function. Multivariable logistic regression analyses examined associations between individual- and community-level social capital and oral frailty risk, as well as the interaction effects of social capital, stratified by SES, on oral frailty risk across SES groups. All models were adjusted for potential confounders, including sex, age, education, household composition, and chronic conditions.

**Results:**

Over three years, 21.6% of participants developed oral frailty risk, with a higher incidence among low-SES participants (24.6%) than among high-SES participants (19.9%). At the higher individual level, higher health-related social capital and social cohesion were significantly associated with lower odds of oral frailty risk across SES groups. Higher civic participation was also associated with lower oral frailty risk across SES groups, with a stronger association observed among low-SES participants. At the community level, higher civic participation was associated with lower oral frailty risk in the overall sample and among low-SES participants. A significant interaction between SES and individual-level civic participation was observed, while no significant interactions were found for other social capital measures.

**Conclusions:**

Individual-level health-related social capital was associated with a lower risk of oral frailty among older adults. Furthermore, the inverse association between civic participation and oral frailty was stronger among individuals with low socioeconomic status, suggesting that promoting social participation may be a promising strategy for reducing socioeconomic inequalities in oral frailty.

## Introduction

One of the most pressing public health challenges in Japan is narrowing the gap between healthy life expectancy and overall life expectancy [1]. According to the World Health Organization (WHO), healthy life expectancy is defined as the average number of years that a person can expect to live in “full health,” if he or she were to pass through the remaining years of life exposed to the sex- and age-specific mortality and morbidity prevailing at the time, for a specific year, in a given country, territory, or geographic area [2]. In other words, it represents the period during which an individual can maintain functional health and independence in daily life, encompassing not only the preservation of physical and psychological functions but also the ability to participate in social activities. In Japan, although both healthy life expectancy and overall life expectancy have been steadily increasing in recent years, a substantial discrepancy persists [3]. Moreover, this gap is closely intertwined with health inequalities that exist across socioeconomic, regional, and gender lines, suggesting that not all population groups benefit equally from advances in medical care and health promotion [4]. It is evident that this gap naturally tends to widen with age and becomes most pronounced in later life. Functional health—including respiratory capacity, muscular strength, and cardiovascular performance—reaches its peak in early adulthood and gradually declines with advancing age. The rate of this decline may vary depending on individual characteristics, living conditions, and behavioral factors [5]. In this sense, health disparities may accumulate across the life course through differences in material resources, health behaviors, and access to supportive social environments. Such cumulative disadvantages may further widen inequalities in functional health outcomes in later life, a process often described as cumulative advantage (or disadvantage). Previous research suggests that cumulative advantage/disadvantage can be defined as the systemic tendency for interindividual divergence in a given characteristic (e.g., money, health, or status) over time [6]. Furthermore, other studies indicate that health disparities are not solely attributable to differences in health status, but are also strongly associated with socioeconomic status (SES). SES is a social determinant of health, encompassing indicators such as income, educational attainment, and occupation, which collectively reflect one’s position within society [7,8]. SES is a structural determinant of health that is not easily modified and plays a fundamental role in shaping health inequalities [9]. A growing body of empirical evidence underscores the substantial impact of SES on older adults’ health. Shibuya et al. (2002) reported that individuals with low income exhibit poorer self-rated health, lower quality of life, and unhealthier lifestyles [10]. Kondo et al. (2009) suggested that residing in regions with large income disparities is associated with elevated risks of premature mortality and poor health [11]. In addition, Murata et al. (2010) found that low-income older adults exhibit poorer health and higher dependency in activities of daily living (ADLs) compared to higher-income counterparts [12]. Ikeda et al. (2019) reported that older adults with lower SES were more likely to experience lower back pain [13]. Wang et al. (2021) demonstrated that lower household income is associated with increased mortality from Alzheimer’s disease and other dementias [14]. Collectively, these findings underscore the clear link between low SES and poor health outcomes among older adults. Because SES in later life is largely determined by long-standing factors that are difficult to modify, identifying effective and equitable strategies to reduce health disparities regardless of SES remains a critical public health priority.

Among older adults, “decline associated with aging” ranks among the leading causes of long-term care needs. This condition, referred to as “frailty,” is a critical target for prevention, as improving frailty can help avert or delay the onset of care dependency. Frailty is defined by the Japan Geriatrics Society (2014) as “a state of increased vulnerability to adverse health outcomes resulting from age-related declines in physiological reserves and functional capacity” [15]. It is widely recognized as a multidimensional geriatric syndrome encompassing physical, psychological, and social domains. Several interrelated subtypes of frailty have been identified, including physical, psychological, social, cognitive, and oral frailty. Among these, oral frailty is characterized as a potentially reversible condition marked by the accumulation of subtle declines in multiple oral functions, such as tooth retention, mastication, swallowing, and speech, which collectively increase the risk of subsequent oral functional impairment [15]. Emerging evidence suggests that oral frailty is closely associated with deterioration in physical, psychological, and social functioning and may accelerate progression toward disability and dependence on long-term care services [16,17]. Furthermore, oral frailty has been linked to reduced social participation, impaired communication resulting from diminished articulation, weakened social connectedness, depressive symptoms, and lower levels of physical activity [18–20]. Taken together, these findings underscore the particular public health significance of oral frailty, highlighting its potential impact not only on oral health but also on healthy ageing, social engagement, and overall quality of life among older adults. According to the Japan Dental Association (2019) [21], early intervention to prevent progression across these stages may help mitigate multiple forms of frailty, thereby contributing to extending healthy life expectancy and narrowing the gap with overall life expectancy.

Income is one of the key indicators of SES. Prior research has demonstrated strong associations between income and oral health. In the United States, low-income individuals are more likely to have poor oral health [22], and low-income older adults in states without Medicaid dental benefits have lower dental visit rates [23]. Similarly, in Japan and the United Kingdom, individuals with lower income and educational attainment have a higher risk of edentulism [24]. These findings indicate an association between SES and oral frailty or its risk factors. However, Japan has a universal health insurance system that covers most dental treatments, allowing older adults to access oral healthcare services with relatively low out-of-pocket costs [25,26]. However, socioeconomic inequalities in oral health persist even in Japan, where universal access to dental care is guaranteed. This suggests that social determinants beyond financial barriers may influence the relationship between SES and oral frailty. Examining these moderating factors may help elucidate the mechanisms through which socioeconomic disadvantage affects oral health outcomes among older adults.

A potential moderating factor in the association between SES and oral frailty risk is social capital. Social capital has increasingly been recognized as an important determinant of health and may be particularly relevant to oral health among older adults. Social capital is generally defined as the resources accessible to individuals and groups through social networks, trust, norms of reciprocity, and civic participation [27]. Previous studies have suggested several pathways through which social capital may influence health outcomes. First, organizational processes within communities may facilitate access to health-related services and promote preventive health behaviors [27]. Second, high levels of social capital are associated with lower psychological distress [28], which has been identified as a risk factor for periodontal disease and other oral health problems [29,30]. Third, trust and collective efficacy may enable residents to improve their living environments and support community-based health promotion activities [31]. These mechanisms suggest that social capital may contribute to maintaining oral function and reducing oral frailty risk in later life. Health-related social capital can be conceptualized at both the individual and community levels. Individual-level social capital refers to resources embedded in an individual’s social relationships, including perceived trust, reciprocity, and social participation. In contrast, community-level social capital reflects the collective characteristics of a community, such as the extent of civic participation, mutual trust, and social cohesion among residents. Because these two levels of social capital may influence health through different mechanisms, examining both levels simultaneously may provide a more comprehensive understanding of their associations with oral frailty risk. Although previous studies have examined associations between social capital and various health outcomes, evidence regarding the relationship between health-related social capital and oral frailty remains limited. Furthermore, little is known about whether these associations differ according to SES. Clarifying such differences is important because older adults with low SES may have fewer material and social resources available to maintain their health, potentially increasing their reliance on social resources derived from community engagement and social relationships. Therefore, this study aimed to examine the associations of individual- and community-level health-related social capital with oral frailty risk among community-dwelling older adults in Japan. In addition, stratified analyses were conducted to explore whether these associations differed between low- and high-SES groups. This study has both theoretical and practical significance. Theoretically, it advances understanding of the role of social capital in oral health by examining both individual- and community-level dimensions. Practically, it may help identify modifiable social resources that can support oral health promotion and contribute to reducing socioeconomic inequalities in healthy aging. We hypothesized that higher levels of individual- and community-level health-related social capital would be associated with a lower risk of oral frailty among community-dwelling older adults. We further explored whether the magnitude of these associations differed according to SES.

## Materials and methods

### Data source

This study used longitudinal data from the Japan Gerontological Evaluation Study (JAGES); [32,33], collected between 2019 and 2022. JAGES is a large-scale, population-based cohort study in Japan investigating social determinants of health among older adults. Self-administered questionnaires were distributed to community-dwelling individuals aged 65 years or older who were physically and cognitively independent.

### Participants

Participants were recruited from 49 municipalities that participated in both the 2019 and 2022 surveys, out of a total of 1,718 municipalities across all 47 prefectures in Japan. Inclusion criteria were age ≥ 65 years and absence of long-term care certification. Municipalities with fewer than 5,000 eligible residents distributed questionnaires to all residents, whereas in municipalities with more than 5,000 eligible residents, participants were selected using stratified random sampling based on the municipal resident registry and social insurance records related to functional independence certification. Baseline data were collected from 66 municipalities between November 2019 and January 2020, and follow-up data from 76 municipalities between November and December 2022. Participants were excluded if they: 1)Did not respond to either the 2019 baseline or 2022 follow-up surveys; 2)Had missing data on baseline characteristics, including sex, age, years of education, chronic diseases, household composition, number of household members, or household income; 3)Had missing data on oral frailty risk items, or were already at risk of oral frailty at baseline, since this study focused on primary prevention; 4)Had missing community-level health-related social capital data at baseline.

### Outcome

#### Oral frailty status at the 2022 survey.

Oral frailty status at baseline and follow-up was assessed using the Oral Frailty Predictive Model developed by Yamamoto et al. (2022) [34]. This model, in the original study, demonstrated a sensitivity of 0.90 and a specificity of 0.66 for classifying oral frailty risk in the testing set. It estimates the probability of oral frailty based on five indicators, all of which are coded as binary variables. (1 = condition present, 0 = condition absent):

1. Age 75–84 years: coded as 1 if participant was 75–84 years old, 0 otherwise.
2. Age ≥ 85 years: coded as 1 if participant was 85 years or older, 0 otherwise.
3. Remaining teeth < 20: coded as 1 if participant had fewer than 20 remaining teeth, 0 otherwise.
4. Impaired chewing function: coded as 1 if participant reported difficulty eating tough foods compared with six months prior, 0 otherwise.
5. Impaired swallowing function: coded as 1 if participant reported recent choking on tea or soup, 0 otherwise.

The oral frailty risk score (*p*) was calculated using the logistic regression equation:


$$\begin{array}{c} p=\frac{\text{exp}(\beta_{\text{0}}+\sum \beta_{i}X_{i})}{\text{1}+\text{exp}(\beta_{\text{0}}+\sum \beta_{i}X_{i})} \end{array}$$

The regression coefficients (0.477, 1.665, 2.563, 2.025, and 2.463) were applied to their respective indicators, with a constant term of −3.983 added. This approach was used to account for the differential impact of each indicator on oral frailty risk, as demonstrated in the original study, thereby ensuring that the score reflects the relative contribution of each factor. Predicted probabilities ranged from approximately 0.0183 to 0.9913, and participants were classified as “Oral frailty risk present” if p ≥ 0.1824, or “Oral frailty risk absent” if p < 0.1824.

In this study, oral frailty was estimated based on self-reported non-clinical indicators and is therefore distinct from a clinical diagnosis. Self-reported measures of dental and oral health status have been reported to show a certain degree of validity in previous studies [35].

### Exposures

#### SES at the 2019 survey.

SES at baseline was assessed using equivalent household income. Equivalent household income was calculated by dividing total household income by the square root of household size. Midpoint values (in 10,000 yen) were assigned to the 15 response categories of total annual household income as follows: 1 = 25, 2 = 75, 3 = 125, 4 = 175, 5 = 225, 6 = 275, 7 = 350, 8 = 450, 9 = 550, 10 = 650, 11 = 750, 12 = 850, 13 = 950, 14 = 1,100, and 15 = 1,200. Participants were categorized into a low-income group (Low-SES; < 2 million yen) and a middle-to-high-income group (High-SES; ≥ 2 million yen). These categories were defined with reference to both the distribution of equivalent household income in the study sample and national income statistics for older adults in Japan. According to the National Survey of Family Income and Expenditure, the average equivalent disposable household income among households with individuals aged 65 years or older was 2.653 million yen in 2018 and 2.794 million yen in 2020. Therefore, the cut-off value of 2.0 million yen represented a level substantially below the national average and was used to identify participants with relatively low socioeconomic status. In this study, due to limitations in the JAGES survey items, some data required to calculate equivalent disposable income were unavailable. Therefore, we calculated equivalent household income and used it as the SES indicator. The Low-SES group was intended to reflect a low-income group below the national average, while the High-SES group reflects a middle-to-high-income group. The threshold of 2 million yen was adopted because it is below the national average for households with individuals aged 65 or older and, based on the distribution in our sample, allowed for a sufficient number of participants in each group [36].

#### Health-related social capital.

Health-Related Social capital was assessed across three domains: civic participation, social cohesion, and reciprocity at individual and community levels at the 2019 Survey.

### Community-level health-related social capital

Community-level social capital in 2019 was assessed using the health-related community social capital scale developed by Saito et al. (2017) [37], comprising three domains: civic participation, social cohesion, and reciprocity. Scores were calculated by aggregating individual responses at the community level. Community-level social capital indicators were calculated using weighted composite scores based on the factor loadings reported in the original JAGES social capital scale development study. The weighting procedure followed the calculation formula provided in the JAGES methodological documentation [38], which reflects the relative contribution of each indicator to the latent social capital construct.

Civic Participation (structural dimension) was assessed via engagement in volunteering, sports, hobbies, study/cultural groups, and skill-teaching activities. Participants were considered engaged if they participated at least once a month. Weighted scores for community-level civic participation were calculated as:


$$\begin{array}{c} \text{0}.\text{6}\times \text{volunteering}+\text{0}.\text{8}\times \text{sports}+\text{0}.\text{9}\times \text{hobby}+\text{0}.\text{7}\times \text{study}/\text{cultural}+\text{0}.\text{5}\times \text{skills teaching} \end{array}$$

The theoretical range of community-level civic participation scores was 0–3.5. Individual-level civic participation scores were calculated by simple summation of the five items, with a score range of 0–5.

Social Cohesion (cognitive dimension) was assessed using three items: community trust, perceived mutual help, and attachment to the area. Community-level scores were weighted as:0.9×trust+0.8×mutual help+0.7×attachment.

The theoretical range of community-level social cohesion scores was 0–2.4. Individual-level social cohesion scores were calculated by simple summation of the three items, with a score range of 0–3.

Reciprocity (support exchange) was assessed via receiving and providing emotional support and receiving instrumental support. Community-level scores were weighted as:


$$\begin{array}{c} \text{0}.\text{8}\times \text{received emotional support}\;\text{0}.\text{7}\times \text{provided emotional support}\;\text{0}.\text{6}\;\text{received instrumental support} \end{array}$$

The theoretical range of community-level reciprocity scores was 0 to 2.1. Individual-level reciprocity scores were calculated by simple summation of the three items, with a score range of 0–3.

Community-level overall health-related social capital was assessed by the sum of community-level civic participation, social cohesion, and reciprocity scores. The total score ranged from 0 to 8.0.

### Individual-level overall health-related social capital

Individual-level overall health-related social capital was assessed by the sum of individual-level civic participation, social cohesion, and reciprocity scores. The total score ranged from 0 to 11.

### Covariates

Analyses controlled for potential confounders: sex (men = 1, women = 2), age, education (< 6, 6–9, 10–12, ≥ 13 years), household composition (living alone = 1, living with others = 0), number of chronic diseases, and SES (for total sample analyses). The number of chronic conditions was calculated by dichotomizing the presence or absence of 17 chronic diseases (hypertension, stroke, heart disease, diabetes, hyperlipidemia, respiratory diseases, gastrointestinal/liver/gallbladder diseases, kidney/prostate diseases, musculoskeletal disorders, trauma, cancer, hematologic/immune disorders, depression, dementia, Parkinson’s disease, eye diseases, and ear diseases) as categorical variables and summing them. The score ranged from 0 to 17. All covariates were measured at baseline (2019).

### Statistical analyses

Descriptive statistics were initially computed to summarize the characteristics of the participants. Baseline characteristics, oral frailty risk at follow-up, and baseline social capital scores were examined for the entire sample, as well as stratified by SES (Low/High). Subsequently, logistic regression analyses were conducted separately for the total sample, the low-SES subgroup, and the high-SES subgroup to investigate the influence of social capital on the association between SES and oral frailty risk. The dependent variable was the presence of oral frailty risk at the follow-up survey (1 = condition present, 0 = condition absent), whereas social capital scores measured at baseline served as the independent variables. All models were adjusted for sex, age, years of education, household composition, and number of chronic diseases. In the analysis of the total sample, SES (income) was additionally included as a covariate. Odds ratios (ORs) with corresponding 95% confidence intervals (CIs) were calculated to quantify the association with oral frailty risk. To mitigate potential multicollinearity among social capital indicators, four types of social capital scores at both the community and individual levels were entered into separate models for each group (total, low-SES, high-SES). Independent-samples t-tests (two-tailed, between-subjects) and chi-square tests were employed to examine statistically significant differences between the low-SES and high-SES groups. To assess the robustness of the results, a sensitivity analysis was conducted using logistic regression with the 2019 survey data. According to the inclusion and exclusion criteria of the present study, the analytic sample consisted of 118,580 participants. The dependent variable was the presence or absence of oral frailty risk. Covariates included sex, age, education years, household composition, and number of chronic diseases. The independent variables were SES and health-related social capital. Furthermore, to formally assess whether the associations between social capital and oral frailty risk varied across SES levels, we conducted logistic regression analyses incorporating interaction terms between SES and each social capital indicator (community-level civic participation, social cohesion, reciprocity, and overall health-related social capital, as well as individual-level civic participation, social cohesion, reciprocity, and overall health-related social capital). Specifically, we fitted models including SES, social capital (SC), an SES × SC interaction term, and relevant covariates (Oral frailty = SES + SC + SES × SC + covariates). These statistical analyses were performed using SPSS version 30.0.0.0 (IBM Corp., Armonk, NY, USA), with a two-sided significance level of 0.05

### Ethics approval and consent to participate

All participants received a written explanation from their respective JAGES municipalities and provided written informed consent before participation. They were informed about the purpose, significance, and methods of the study; the voluntary nature of their participation; the use and publication of anonymized data; and their right to withdraw at any time without penalty. These details were explained both in writing and orally by the principal investigator, and consent was obtained through signed consent forms.

This study targeted adults aged 65 years and older; no minors were included. All data were fully anonymized, and participants explicitly provided written consent for the use of their anonymized data for research purposes. The study was conducted in accordance with the Declaration of Helsinki (1964) and its subsequent amendments, as well as the ethical guidelines for life sciences and medical research involving human participants established by the Ministry of Health, Labour and Welfare of Japan. Ethical approval was obtained from the Institutional Review Board of Hokkaido University (Certificate No. 25–32; July 10, 2025).

## Results

**Fig 1:**
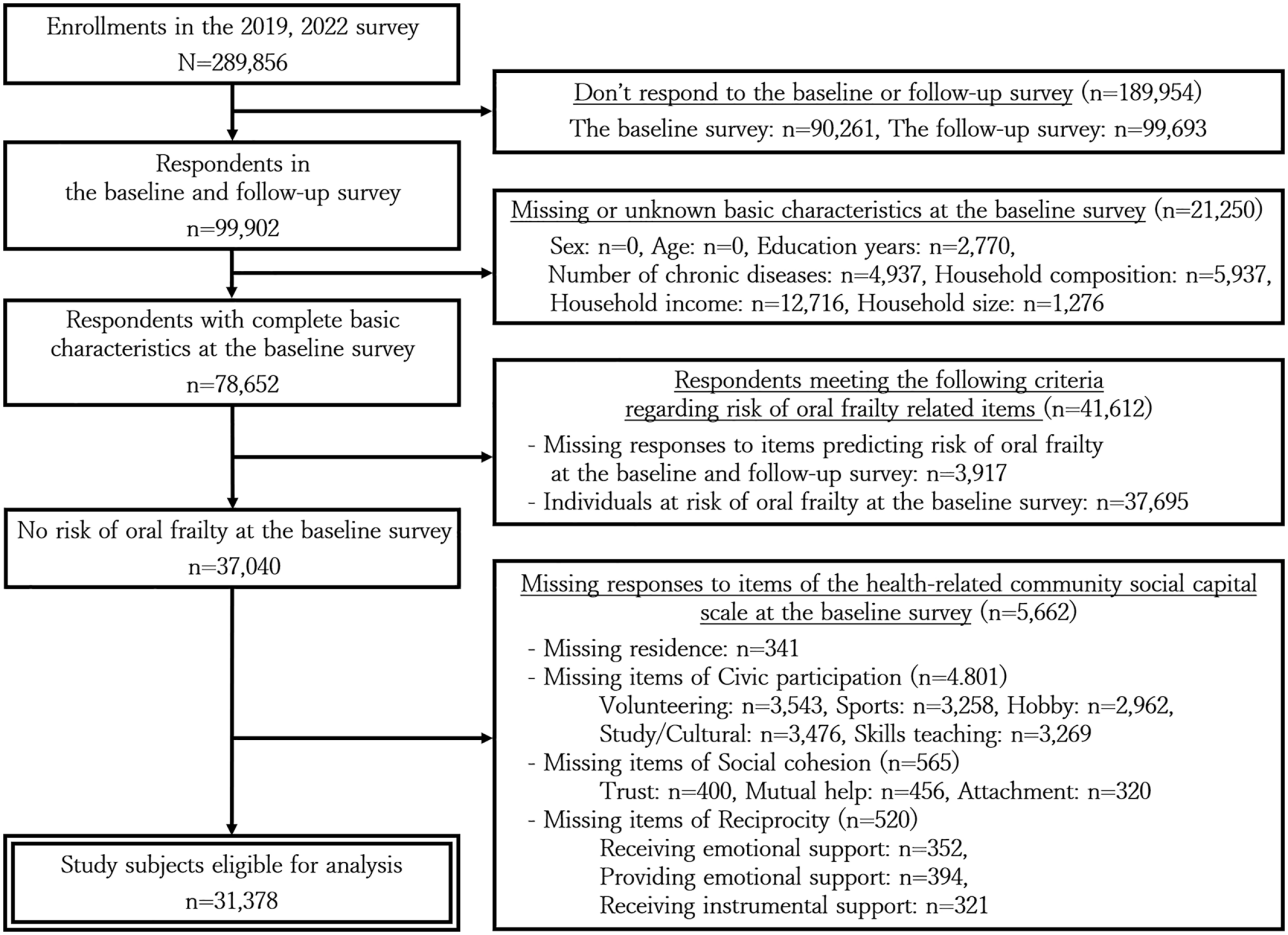
Flowchart of study participants.

A total of 99,902 individuals completed both the 2019 baseline and the 2022 follow-up surveys. After applying the exclusion criteria, the final analytic sample consisted of 31,378 participants (Fig 1). Additionally, the exclusion criteria included 37,695 participants who had been identified as having a risk of oral frailty.

Table 1 summarizes the incidence of oral frailty risk during the three-year follow-up and baseline characteristics of the analytic sample participants by SES. During the three-year follow-up period, the overall incidence of oral frailty risk was 21.6%. Among those with oral frailty risk, 24,587 individuals (78.4%) were from the total sample (n = 31,378), 8,796 (75.4%) from the low-SES group (n = 11,662), and 15,791 (80.1%) from the high-SES group (n = 19,716). The mean age of participants was 71.7 years (SD = 4.9, *p* < 0.001), and 37.2% belonged to the low-SES group. S1 Table compares the baseline characteristics of the analytic sample and excluded participants, including individuals with missing data. The excluded participants were generally older and less educated than those included in the analytic sample. Specifically, the mean age of excluded participants was 75.3 years, compared with 71.7 years in the analytic sample, and the proportion with ≥13 years of education was lower among excluded participants (17.4%) than among the analytic sample (40.4%).

**Table 1:** The risk of oral frailty and characteristics of the participants.

	Overall(n = 31,378)	Low-SES(n = 11,662)	High-SES(n = 19,716)	*p* value
**The risk of oral frailty, n (%)**				<0.001**
**Absence**	**24,587(78.4)**	**8,796(75.4)**	**15,791(80.1)**	
**Presence**	**6,791(21.6)**	**2,866(24.6)**	**3,925(19.9)**	
**Sex, n (%)**				<0.001**
**men**	**15,824(50.4)**	**5,598(48.0)**	**10,226(51.9)**	
**women**	**15,554(49.6)**	**6,064(52.0)**	**9,490(48.1)**	
**Age, mean(±SD)**	**71.7(±4.9)**	**72.2(±4.9)**	**71.5(±4.8)**	<0.001**
**Education, n (%)**				<0.001**
**< 6 years**	**23(0.1)**	**15(0.1)**	**8(0.0)**	
**6–9 years**	**4,357(13.9)**	**2,401(20.6)**	**1,956(9.9)**	
**10–12 years**	**14,335(45.7)**	**5,618(48.2)**	**8,717(44.2)**	
**≥ 13 years**	**12,663(40.4)**	**3,628(31.1)**	**9,035(45.8)**	
**Living alone, n (%)**				<0.001**
**No**	**27,562(87.8)**	**9,723(83.4)**	**17,839(90.5)**	
**Yes**	**3,816(12.2)**	**1,939(16.6)**	**1,877(9.5)**	
**Chronic disease, mean(±SD)**	**1.4(±1.2)**	**1.5(±1.2)**	**1.4(±1.2)**	<0.001**

*p < 0.05, **p < 0.001

Table 2 presents the social capital scores at both the community and individual levels, stratified by SES. Significant differences between low-SES and high-SES groups were observed across all measures of social capital—including civic participation, social cohesion, reciprocity, and overall health-rated social capital—at both the community and individual levels.

**Table 2:** Social capital scores at the community and individual levels by SES.

		Overall (n = 31,378)			Low-SES (n = 11,662)			High-SES (n = 19,716)			*p* value
		Min	Max	Mean (±SD)	Min	Max	Mean (±SD)	Min	Max	Mean (±SD)	
**Community-level**	**Civic participation**	**0.00**	**1.83**	**0.76 (±.20)**	**0.00**	**1.83**	**0.74 (±.20)**	**0.00**	**1.83**	**0.77 (±.20)**	<0.001 **
	**Social cohesion**	**0.40**	**2.40**	**1.70 (±.17)**	**0.40**	**2.40**	**1.70 (±.18)**	**0.40**	**2.40**	**1.71 (±.17)**	<0.001 **
	**Reciprocity**	**1.17**	**2.10**	**2.01 (±.06)**	**1.17**	**2.10**	**2.01 (±.06)**	**1.17**	**2.10**	**2.01 (±.06)**	0.013*
	**Overall health-related social capital**	**2.32**	**5.79**	**4.48 (±.30)**	**2.32**	**5.79**	**4.45 (±.30)**	**2.32**	**5.79**	**4.50 (±.30)**	<0.001 **
**Individual-level**	**Civic participation**	**0**	**5**	**0.98 (±1.17)**	**0**	**5**	**0.87 (±1.14)**	**0**	**5**	**1.05 (±1.18)**	<0.001 **
	**Social cohesion**	**0**	**3**	**2.14 (±1.04)**	**0**	**3**	**2.03 (±1.09)**	**0**	**3**	**2.20 (±1.00)**	<0.001 **
	**Reciprocity**	**0**	**3**	**2.88 (±.45)**	**0**	**3**	**2.83 (±.53)**	**0**	**3**	**2.90 (±.39)**	**<0.001**
	**Overall health-related social capital**	**0**	**11**	**6.00 (±1.79)**	**0**	**11**	**5.73 (±1.85)**	**0**	**11**	**6.15 (±1.73)**	<0.001 **

*p < 0.05, **p < 0.001

Table 3 details the associations between civic participation scores and incident oral frailty risk at both community and individual levels. At the community level, higher civic participation was significantly associated with reduced odds of developing oral frailty risk in the overall sample (OR = 0.833, 95% CI: 0.723–0.961) and in the low-SES group (OR = 0.781, 95% CI: 0.625–0.976), after adjustment for covariates. At the individual level, higher civic participation scores were strongly and significantly associated with lower odds of developing oral frailty risk across the overall sample (OR = 0.949, 95% CI: 0.925–0.973) and the low-SES group (OR = 0.920, 95% CI: 0.884–0.959). A similar, though weaker, association was observed in the high-SES group (OR = 0.967, 95% CI: 0.936–0.998). Furthermore, a significant interaction was observed between SES and individual-level civic participation in relation to oral frailty (OR for interaction = 0.980, 95% CI = 0.893–0.966, p = 0.012), indicating that the association between SES and oral frailty varied according to the level of civic participation.

**Table 3:** Effects of community- and individual-level civic participation on oral frailty risk.

	Overall			Low-SES			High-SES		
	OR	95%CI	*p* value	OR	95%CI	*p* value	OR	95%CI	*p* value
**Sex**									
**Men**	**ref**			**ref**			**ref**		
**Women**	**0.937**	**0.886 - 0.992**	**0.025**	**0.889**	**0.813 - 0.971**	**0.009**	**0.968**	**0.899 - 1.042**	**0.387**
**Age**	**1.092**	**1.086 - 1.098**	**<0.001**	**1.082**	**1.073 - 1.092**	**<0.001**	**1.099**	**1.091 - 1.107**	<0.001 **
**Education**	**0.875**	**0.840 - 0.912**	**<0.001**	**0.882**	**0.829 - 0.937**	**<0.001**	**0.868**	**0.822 - 0.917**	<0.001**
**Household composition**									
**Living with others**	**ref**			**ref**			**ref**		
**Living alone**	**1.010**	**0.929 - 1.098**	**0.809**	**1.070**	**0.952 - 1.202**	**0.254**	**0.956**	**0.847 - 1.079**	**0.466**
**Chronic diseases**	**1.095**	**1.070 - 1.121**	**<0.001**	**1.093**	**1.054 - 1.133**	**<0.001**	**1.097**	**1.065 − 1,131**	<0.001**
**SES**									
**High-SES**	**ref**								
**Low-SES**	**1.183**	**1.117 - 1.253**	**<0.001**	–	–	–	–	–	–
**Community-level Civic participation score**	**0.833**	**0.723 - 0.961**	**0.012**	**0.781**	**0.625 - 0.976**	**0.030**	**0.872**	**0.725 - 1.049**	**0.146**
**Individual-level Civic participation score**	**0.949**	**0.925 - 0.973**	**<0.001**	**0.920**	**0.884 - 0.959**	**<0.001**	**0.967**	**0.936 - 0.998**	0.037*
**SES×Community-level Civic participation**	**0.815**	**0.616-1.077**	**0.150**						
**SES×Individual-level Civic participation**	**0.980**	**0.893-0.966**	**0.012**						

*p < 0.05, **p < 0.001

Table 4 presents the associations between social cohesion scores and incident oral frailty risk. No significant associations were observed at the community level in any group. At the individual level, however, higher social cohesion scores were robustly and significantly associated with reduced odds of developing oral frailty risk across all groups: overall (OR = 0.925, 95% CI: 0.901–0.951), low-SES (OR = 0.925, 95% CI: 0.889–0.963), and high-SES (OR = 0.927, 95% CI: 0.894–0.961), after controlling for covariates.　The interaction terms between SES and community-level social cohesion (OR = 1.016, 95% CI = 0.739–1.396, p = 0.923) and between SES and individual-level social cohesion (OR = 0.993, 95% CI = 0.899–1.165, p = 0.790) were not statistically significant in relation to oral frailty.

**Table 4:** Effects of community and individual-level social cohesion on oral frailty risk.

	Overall			Low-SES			High-SES		
	OR	95%CI	*p* value	OR	95%CI	*p* value	OR	95%CI	*p* value
**Sex**									
**Men**	**ref**			**ref**			**ref**		
**Women**	**0.914**	**0.865 - 0.967**	**0.002**	**0.860**	**0.788 - 0.940**	**<0.001**	**0.949**	**0.883 - 1.021**	**0.161**
**Age**	**1.091**	**1.085 - 1.097**	**<0.001**	**1.080**	**1.070 - 1.089**	**<0.001**	**1.098**	**1.090 - 1.106**	<0.001**
**Education**	**0.861**	**0.827 - 0.897**	**<0.001**	**0.862**	**0.811 - 0.915**	**<0.001**	**0.859**	**0.814 - 0.917**	<0.001**
**Household composition**									
**Living with others**	**ref**			**ref**			**ref**		
**Living alone**	**1.000**	**0.920 - 1.087**	**1.000**	**1.060**	**0.944 - 1.191**	**0.326**	**0.946**	**0.838 - 1.067**	**0.365**
**Chronic diseases**	**1.096**	**1.071 - 1.121**	**<0.001**	**1.096**	**1.057 - 1.136**	**<0.001**	**1.096**	**1.064 - 1.130**	<0.001**
**SES**									
**High-SES**	**ref**								
**Low-SES**	**1.186**	**1.120 - 1.256**	**<0.001**	**–**	**–**	**–**	**–**	**–**	**–**
**Community-level Social cohesion score**	**1.111**	**0.946 - 1.305**	**0.198**	**1.124**	**0.879 - 1.438**	**0.351**	**1.099**	**0.889 - 1.359**	**0.385**
**Individual-level Social cohesion score**	**0.925**	**0.901 - 0.951**	**<0.001**	**0.925**	**0.889 - 0.963**	**<0.001**	**0.927**	**0.894 - 0.961**	<0.001**
**SES×Community-level Social cohesion**	**1.016**	**0.739-1.396**	**0.923**						
**SES×Individual-level Social cohesion**	**0.993**	**0.899-1.165**	**0.790**						

*p < 0.05, **p < 0.001

Table 5 summarizes the effects of reciprocity scores on incident oral frailty risk. Community-level reciprocity was not significantly associated with the incidence of oral frailty risk in any subgroup. At the individual level, higher reciprocity scores were significantly associated with lower odds of developing oral frailty risk in the overall sample following covariate adjustment (OR = 0.931, 95% CI: 0.875–0.991). The interaction terms between SES and community-level reciprocity (OR = 0.628, 95% CI = 0.239–1.651, p = 0.346) and between SES and individual-level reciprocity (OR = 0.953, 95% CI = 0.880–1.054, p = 0.431) were not statistically significant.

**Table 5:** Effects of community and individual-level reciprocity on oral frailty risk.

	Overall			Low-SES			High-SES		
	OR	95%CI	*p* value	OR	95%CI	*p* value	OR	95%CI	*p* value
**Sex**									
**Men**	**ref**			**ref**			**ref**		
**Women**	**0.925**	**0.874 - 0.978**	**0.006**	**0.872**	**0.798 - 0.954**	**0.003**	**0.957**	**0.890 - 1.030**	**0.242**
**Age**	**1.089**	**1.083 - 1.095**	**<0.001**	**1.078**	**1.069 - 1.088**	**<0.001**	**1.097**	**1.089 - 1.105**	<0.001**
**Education**	**0.858**	**0.824 - 0.893**	**<0.001**	**0.857**	**0.807 - 0.911**	**<0.001**	**0.856**	**0.812 - 0.903**	<0.001**
**Household composition**									
**Living with others**	**ref**			**ref**			**ref**		
**Living alone**	**0.989**	**0.907 - 1.078**	**0.797**	**1.043**	**0.923 - 1.178**	**0.499**	**0.942**	**0.831 - 1.067**	**0.346**
**Chronic diseases**	**1.099**	**1.074 - 1.124**	**<0.001**	**1.098**	**1.059 - 1.138**	**<0.001**	**1.100**	**1.067 - 1.133**	<0.001**
**SES**									
**High-SES**	**ref**								
**Low-SES**	**1.194**	**1.127 - 1.264**	**<0.001**	–	–	–	–	–	–
**Community-level Reciprocity score**	**1.356**	**0.833 - 2.207**	**0.220**	**1.089**	**0.514 - 2.308**	**0.824**	**1.591**	**0.839 - 3.017**	**0.155**
**Individual-level Reciprocity score**	**0.931**	**0.875 - 0.991**	**0.025**	**0.933**	**0.857 - 1.015**	**0.106**	**0.941**	**0.857 - 1.034**	**0.206**
**SES×Community-level Reciprocity**	**0.628**	**0.239-1.651**	**0.346**						
**SES×Individual-level Reciprocity**	**0.953**	**0.880-1.054**	**0.431**						

*p < 0.05, **p < 0.001

Finally, Table 6 illustrates the associations between overall health-related social capital scores and incident oral frailty risk. No significant associations were found at the community level in any group. In contrast, higher individual-level health-related social capital scores were strongly and significantly associated with reduced odds of developing oral frailty risk across all groups: overall (OR = 0.947, 95% CI: 0.932–0.963), low-SES (OR = 0.938, 95% CI: 0.915–0.961), and high-SES (OR = 0.956, 95% CI: 0.935–0.976), after adjusting for covariates. The interaction terms between SES and community-level overall health-related social capital score (OR = 0.909, 95% CI = 0.841–1.093) and between SES and individual-level score (OR = 0.971, 95% CI = 0.942–1.002) were not statistically significant.

**Table 6:** Effects of community and individual-level health-related social capital on oral frailty risk.

	Overall			Low-SES			High-SES		
	OR	95%CI	*p* value	OR	95%CI	*p* value	OR	95%CI	*p* value
**Sex**									
**Men**	**ref**			**ref**			**ref**		
**Women**	**0.942**	**0.890 - 0.996**	**0.037**	**0.893**	**0.817 - 0.976**	**0.013**	**0.973**	**0.904 - 1.047**	**0.465**
**Age**	**1.092**	**1.086 - 1.098**	**<0.001**	**1.082**	**1.073 - 1.092**	**<0.001**	**1.099**	**1.091 - 1.107**	<0.001**
**Education**	**0.874**	**0.839 - 0.910**	**<0.001**	**0.876**	**0.825 - 0.931**	**<0.001**	**0.869**	**0.823 - 0.918**	<0.001**
**Household composition**									
**Living with others**	**ref**			**ref**			**ref**		
**Living alone**	**0.980**	**0.901 - 1.066**	**0.640**	**1.028**	**0.914 - 1.156**	**0.644**	**0.934**	**0.827 - 1.055**	**0.272**
**Chronic diseases**	**1.093**	**1.068 - 1.118**	**<0.001**	**1.091**	**1.052 - 1.131**	**<0.001**	**1.095**	**1.062 - 1.128**	<0.001**
**SES**									
**High-SES**	**ref**								
**Low-SES**	**1.174**	**1.109 - 1.244**	**<0.001**	–	–	–	–	–	–
**Community-level Overall Health-related social capital score**	**0.969**	**0.883 - 1.063**	**0.503**	**0.942**	**0.816 - 1.088**	**0.417**	**0.987**	**0.874 - 1.115**	**0.835**
**Individual-level Overall Health-related social capital score**	**0.947**	**0.932 - 0.963**	**<0.001**	**0.938**	**0.915 - 0.961**	**<0.001**	**0.956**	**0.935 - 0.976**	<0.001**
**SES×Community-level Overall Health-related social capital score**	**0.909**	**0.841-1.093**	**0.311**						
**SES×Individual-level Overall Health-related social capital score**	**0,971**	**0.942-1.002**	**0.057**						

*p < 0.05, **p < 0.001

The sensitivity analysis revealed significant associations in both the low- and high-SES groups for community-level civic participation (low SES: OR = 0.249, 95% CI: 0.223–0.278; high SES: OR = 0.334, 95% CI: 0.301–0.372), reciprocity (low SES: OR = 0.427, 95% CI: 0.290–0.629; high SES: OR = 0.512, 95% CI: 0.349–0.751), and overall social capital (low SES: OR = 0.544, 95% CI: 0.508–0.583; high SES: OR = 0.616, 95% CI: 0.576–0.659), as well as for individual-level civic participation (low SES: OR = 0.864, 95% CI: 0.849–0.878; high SES: OR = 0.879, 95% CI: 0.866–0.893), social cohesion (low SES: OR = 0.925, 95% CI: 0.911–0.940; high SES: OR = 0.931, 95% CI: 0.916–0.947), reciprocity (low SES: OR = 0.843, 95% CI: 0.817–0.869; high SES: OR = 0.885, 95% CI: 0.851–0.920), and overall social capital (low SES: OR = 0.910, 95% CI: 0.901–0.919; high SES: OR = 0.917, 95% CI: 0.908–0.926) (S1 Table).

## Discussion

The main findings of this study can be summarized as follows. First, over the three-year follow-up period, the incidence of oral frailty among community-dwelling, independent older adults was 21.6%, with a higher incidence observed in the low socioeconomic status (SES) group (24.6%) than in the high SES group (19.9%). Second, after adjustment for key demographic and health-related covariates, individual-level social capital showed more consistent associations with oral frailty than community-level social capital. Specifically, individual-level health-related social capital, civic engagement, reciprocity, and social cohesion were inversely associated with oral frailty in the overall sample as well as in both SES groups. Third, and most notably, among the eight social capital indicators examined, only individual-level civic engagement significantly modified the association between SES and oral frailty. The inverse association between civic engagement and oral frailty was stronger among older adults with lower SES than among those with higher SES. This finding suggests that although social capital is generally associated with oral frailty regardless of SES, civic engagement may play a particularly important role in reducing socioeconomic disparities in oral frailty among older adults.

The finding that individual-level social capital was more consistently associated with oral frailty than community-level social capital suggests that personal social relationships and direct social engagement may be more influential determinants of oral health trajectories in later life than broader community characteristics. Previous studies have proposed several pathways through which social capital may influence health, including the dissemination of health-related information, reinforcement of health-promoting norms and behaviours, provision of social support, and facilitation of access to community resources [21,39]. Individual-level social capital may more directly reflect these mechanisms because it captures an individual’s actual participation in social activities, perceived trust, and access to supportive social networks. Through these pathways, individual-level social capital may influence health-related behaviours, psychological well-being, and access to health information and services, thereby contributing to the maintenance of oral function and the prevention of oral frailty. In contrast, community-level social capital represents contextual social environments that may not necessarily translate into meaningful social interactions or support for every individual. Therefore, its influence on oral frailty may be less direct than that of individual-level social capital. Among the various dimensions of social capital, civic engagement appeared to be particularly important, as it was the only indicator that significantly modified the association between SES and oral frailty. Civic engagement may reflect the extent to which communities provide opportunities for active social participation, thereby influencing oral health through increased physical activity, social interaction, and exposure to health-related information [40]. This finding suggests that civic engagement may play a unique role in mitigating socioeconomic disparities in oral frailty among older adults.

In contrast, no significant interactions with SES were observed for social cohesion or reciprocity at either the community or individual level. These findings suggest that the relationship between social capital and oral frailty may differ according to the specific dimension of social capital being considered. Although social capital is often conceptualized as a multidimensional construct, its components may operate through distinct mechanisms and exert different influences on health outcomes. Civic engagement primarily reflects active participation in social and community activities, whereas social cohesion and reciprocity are more closely related to perceptions of trust, belonging, and mutual support. The present findings suggest that these dimensions of social capital may not contribute equally to the prevention of oral frailty or to the reduction of socioeconomic disparities in oral health among older adults.

These findings have important public health implications. The observed associations between individual-level social capital and oral frailty suggest that efforts to promote social participation and strengthen social connections may contribute to oral frailty prevention among older adults. Furthermore, the significant interaction between civic engagement and SES highlights the potential value of community-based strategies that encourage social participation among socioeconomically disadvantaged older adults. Such approaches may help reduce socioeconomic inequalities in oral health while supporting healthy ageing [41].

This study has several limitations. First, oral frailty was assessed using self-reported non-clinical indicators rather than a clinical diagnosis. Although previous studies have demonstrated the validity of self-reported measures of oral health status [35], the outcome used in this study represents a population-based estimate of oral frailty risk rather than a clinically confirmed diagnosis. Accordingly, the findings should be interpreted as reflecting relative differences in oral frailty risk rather than definitive clinical diagnoses. Second, although we adjusted for a range of demographic and health-related covariates, residual confounding cannot be entirely excluded. Unmeasured factors, such as access to dental care, dietary habits, and other health-related behaviours, may have influenced the observed associations. In addition, excluded participants were generally older and less educated than those included in the analytic sample. Therefore, selection bias resulting from participant exclusion cannot be ruled out, and the findings may be less generalisable to older adults with poorer socioeconomic profiles. Third, although the JAGES dataset and municipality-level social capital indicators are well suited to multilevel analyses, conventional regression models were employed to examine the associations and effect modification among socioeconomic status, social capital, and oral frailty. Consequently, clustering of individuals within municipalities may not have been fully accounted for, potentially reducing the precision of contextual effect estimates. In addition, community-level social capital indicators were derived from weighted composite scores based on factor loadings reported in a previous JAGES study rather than from factor scores estimated using the present dataset. As a result, measurement error and uncertainty in the latent factor structure may not have been fully captured. Finally, the study period overlapped with the COVID-19 pandemic, which substantially altered opportunities for social interaction and community participation among older adults. The pandemic may therefore have affected both social capital and oral frailty risk, potentially influencing the magnitude of the observed associations. Furthermore, if individuals who experienced severe health consequences during the pandemic were less likely to participate in the follow-up survey, survivor bias may have occurred.

## Conclusions

Individual-level health-related social capital was associated with a lower risk of oral frailty among older adults. Furthermore, the inverse association between civic participation and oral frailty was stronger among individuals with low socioeconomic status, suggesting that social participation may play an important role in mitigating socioeconomic disparities in oral frailty. These findings highlight the potential value of interventions that promote social engagement and civic participation as part of public health strategies to support equitable and healthy ageing.

## Supporting information

S1 Table Baseline characteristics between included and excluded participants. (XLSX)
