# Supplementary material for: Associations between health-related social capital and oral frailty risk among older adults: A stratified analysis by socioeconomic status in the JAGES longitudinal study
Source: PLoS One. 2026 Jul 24;21(7):e0353850. doi: 10.1371/journal.pone.0353850 (PMC13399358; doi:10.1371/journal.pone.0353850)
Supplement: Attachment — Submitted filename: 260319Response to Reviewers.docx [file pone.0353850.s003.docx]

**To the Editor**

1)

It is unclear why the authors concluded that individual‑level social capital mitigates SES‑related disparities in oral frailty risk. Which specific results support this conclusion? In the Introduction, the manuscript refers to “mediation,” and the title also implies a mediating effect. However, the study does not appear to assess mediation in a systematic manner, such as through mediation analysis. Please clarify how the authors evaluated a mediating effect and which findings indicate that social capital mediates, rather than moderates, the association between SES and oral frailty risk.

***⇒ Thank you very much for your insightful comment. We sincerely apologize for not clearly describing in the original manuscript how the mediating effect was evaluated and which results support the conclusion that individual-level social capital mediates the association between SES and oral frailty risk.***

***In response to your valuable suggestion, we conducted an additional mediation analysis using structural equation modeling (SEM) to systematically evaluate the mediating role of individual-level social capital in the association between SES and oral frailty risk. Based on the results of this analysis, we have revised the manuscript accordingly.***

***Specifically, the analytical procedure for the mediation analysis has been added to the Methods section (Lines 329-333). The results of the mediation analysis have been presented in the Results section (Lines 422–430, 444-452), and the estimated mediation pathways are illustrated in Figure 2-9.***

***We appreciate this important comment, which helped us clarify the analytical framework and strengthen the rigor of the manuscript.***

***Methods section (Lines 329-333): “For the social capital variables that showed significant associations with oral frailty risk in the low-SES group in the logistic regression analyses, structural equation modeling (SEM) was conducted for both the low-SES and high-SES groups to analyze the mediating structure. Model fit was evaluated using the chi-square test, GFI, CFI, and RMSEA.”***

***Results section (Lines 422–430): “Fig 2–5 present the results of SEM examining the mediating role of social capital indicators that showed significant associations in the low-SES group. Fig 2 represents the mediating structure of Community-level Civic participation (χ²(4) = 109.006, GFI = .996, CFI = .854, RMSEA = .047), Fig 3 represents the mediating structure of Individual-level Civic participation (χ²(1) = 2.240, GFI = 1.000, CFI = .998, RMSEA = .010), Fig 4 represents the mediating structure of Individual-level Social cohesion (χ²(1) = 1.721, GFI = 1.000, CFI = .998, RMSEA = .008), and Fig 5 represents the mediating structure of Individual-level Overall Social Capital (χ²(1) = .128, GFI = 1.000, CFI = 1.000, RMSEA = .000). These findings are consistent with the associations observed in the logistic regression analyses.”***

***(Lines 444-452): “Fig 6–9 present the results of SEM in the high-SES group, corresponding to the Social capital indicators examined in the low-SES group. Fig 6 represents the mediating structure of Community-level Civic participation (χ²(1) = 3.320, GFI = 1.000, CFI = .997, RMSEA = .011), Fig 7 represents the mediating structure of Individual-level Civic participation (χ²(1) = 9.122, GFI = 1.000, CFI = .988, RMSEA = .020), Fig 8 represents the mediating structure of Individual-level Social cohesion (χ²(1) = .151, GFI = 1.000, CFI = 1.000, RMSEA = .000), and Fig 9 represents the mediating structure of Individual-level Overall Social Capital (χ²(1) = 3.441, GFI = 1.000, CFI = .996, RMSEA = .011). These findings are consistent with the associations observed in the logistic regression analyses.”***

2)

The meaning of the reported sensitivity (0.94) and specificity (0.67) on page 9, line 205, is unclear. Sensitivity and specificity of what measure or classification? Additionally, it is not clear why the oral frailty risk score was calculated using a logistic regression equation rather than using the raw score. Please explain the rationale for this approach.

***⇒ Thank you very much for your helpful comment. We apologize that the description regarding the reported sensitivity and specificity was unclear in the original manuscript.***

***The sensitivity (0.94) and specificity (0.67) refer to the predictive performance of the Oral Frailty Predictive Model developed by Yamamoto et al. (2022) for identifying individuals at risk of oral frailty. Specifically, these values represent the model’s ability to correctly classify participants as “at risk of oral frailty” (p ≥ 0.1824) or “not at risk” (p < 0.1824) according to the optimal probability cutoff reported in the original study. We have revised the manuscript to clarify that these indices describe the diagnostic performance of the model for oral frailty risk classification. In addition, based on the values reported in the cited study, the sensitivity and specificity have been corrected to 0.90 and 0.66, respectively, in the revised manuscript.***

***(Lines 218-221): “This model, in the original study, demonstrated a sensitivity of 0.90 and a specificity of 0.66 for classifying oral frailty risk in the testing set. It estimates the probability of oral frailty based on five indicators, all of which are coded as binary variable”***

***⇒ Regarding the use of the logistic regression equation rather than a simple raw score, the Oral Frailty Predictive Model was originally developed using logistic regression analysis, in which each indicator was assigned a coefficient reflecting its relative contribution to oral frailty risk. The predicted probability derived from this equation therefore, represents a weighted combination of the five indicators, providing a more accurate estimation of oral frailty risk than an unweighted raw score. In our study, we applied the published regression coefficients and cutoff value as reported in the original study. We followed the original algorithm without modification to ensure comparability with previous studies.　To improve clarity, we have revised the description in the Methods section (Lines 231-234) to explicitly explain the meaning of the sensitivity and specificity and the rationale for calculating the oral frailty risk score using the logistic regression–based probability.　We appreciate this important comment, which helped us clarify the description of the oral frailty risk assessment method.***

***(Lines 231-234): “The regression coefficients (0.477, 1.665, 2.563, 2.025, and 2.463) were applied to their respective indicators, with a constant term of -3.983 added. This approach was used to account for the differential impact of each indicator on oral frailty risk, as demonstrated in the original study, thereby ensuring that the score reflects the relative contribution of each factor.”***

3)

There are instances of overstatement and insufficient description. For example, line 412 states that “In the high‑SES group, community‑level social capital had no significant effect on oral frailty.” However, this appears to apply only to the civic participation score. In the low‑SES group, the social cohesion score also shows no significant effect on oral frailty. In the original manuscript, since the results derived from multiple measurement items are being interpreted conveniently, please clarify the interpretation of the results.

***⇒ Thank you very much for your careful reading of our manuscript and for your insightful comments. We sincerely appreciate your important observation regarding the potential overstatement and the need for clearer interpretation of the results. In response to this comment, we have revised the manuscript to clarify the interpretation of the findings. Specifically, we have distinguished the results by each component of both community-level and individual-level social capital within the high-SES and low-SES groups.***

***(Lines 499-502):* In the high-SES group, a one-point increase in individual-level civic participation, social cohesion, and health-related social capital corresponded to 3.3%, 7.3%, and 4.4%, lower odds of incident oral frailty risk, respectively, while community-level social capital and individual-level reciprocity showed no significant associations.**

**(Lines 510-511): At the community level, only civic participation among low-SES individuals exhibited a significant effect.**

***(Lines 527-528): “In the high-SES group, community-level civic participation, social cohesion, reciprocity, and overall health-related social capital had no significant effect on oral frailty risk.”***

**To Reviewer 1**

1)

This longitudinal cohort study examined the mediating role of health-related social capital in the relationship between socioeconomic status (SES) and oral frailty risk among community-dwelling older adults in Japan, using data from the Japan Gerontological Evaluation Study (JAGES). The study is well-structured and based on a large, nationally representative dataset, and the topic is timely. Below are suggestions to improve clarity, transparency, and scientific validity, particularly with respect to observational study reporting guidelines.

First, the paper mostly aligns with STROBE reporting standards, but a few checklist items could be improved for completeness and transparency. The abstract includes a clear summary of the design and findings, though the specific mention of the longitudinal nature of the cohort could be made more explicit. A flow diagram is appropriately provided (Figure on page 35), and descriptive statistics and model outputs are clearly presented (Tables 1–7, pages 14–18). However, information on missing data for each variable (STROBE Item 14b) is not fully reported. It would be helpful to specify the extent of missingness for key variables and how they were handled, particularly given the large initial sample and substantial exclusions during follow-up.

***⇒ Thank you for your comment. In accordance with STROBE Item 1, we have revised the abstract as follows. (Lines 29-31)***

***(Lines 29-31): “This study was a longitudinal cohort study of community-dwelling older adults in Japan, aiming to examine the mediating effects of community- and individual-level social capital on the relationship between SES and oral frailty risk.”***

***⇒ Regarding STROBE Item 14b, we have added to Fig. 1 the number of participants excluded according to each exclusion criterion for the following items. The numbers are presented in accordance with the sequence of the study flowchart.***

***- First exclusion: Don’t respond to the baseline survey (n=90,261), Don’t respond to the follow-up survey (n=99,693),***

***- Second exclusion: Sex (n=0), Age (n=0), Education years (n=2,770), Number of chronic diseases (n=4,937), Household composition (n=5,937), Household income (n=12,716), Household size (n=1,276),***

***- Third exclusion: Oral Frailty Predictive Model items at baseline or follow-up (n=3,917), Individuals with oral frailty at the baseline survey (n=37,695),***

***- Fourth exclusion: Missing residence (n=341), Missing items of Civic participation (n=4.801), Volunteering (n=3,543), Sports (n=3,258), Hobby (n=2,962), Study/Cultural (n=3,476), Skills teaching (n=3,269), Missing items of Social cohesion (n=565), Trust (n=400), Mutual help (n=456), Attachment (n=320), Missing items of Reciprocity (n=520), Receiving emotional support (n=352), Providing emotional support (n=394), Receiving instrumental support (n=321)***

2)

Second, the use of a validated predictive model to estimate oral frailty risk at follow-up is appropriate, but the model itself is based on non-clinical indicators (e.g., swallowing function, chewing difficulty). The distinction between predicted risk and actual clinical diagnosis should be more clearly emphasized, and the outcome measure described consistently throughout the manuscript. This is important for construct validity.

***⇒ Thank you very much for your valuable comment regarding the definition and interpretation of the oral frailty outcome measure.　In this study, oral frailty at baseline and follow-up was assessed using the Oral Frailty Predictive Model developed by Yamamoto et al. (2022). As the reviewer correctly noted, this model does not provide a clinical diagnosis of oral frailty; rather, it estimates the probability of oral frailty risk. The model incorporates five indicators—including age group, number of remaining teeth, self-reported masticatory difficulty, and swallowing difficulty—and calculates the predicted probability using a logistic regression equation. Based on the cutoff value proposed in the original study, participants are classified as “at risk of oral frailty” (p ≥ 0.1824) or “not at risk” (p < 0.1824).　We agree that it is important to clearly distinguish between predicted risk and clinical diagnosis. Accordingly, we revised the manuscript to consistently clarify that the outcome in this study represents predicted oral frailty risk, rather than a clinical diagnosis. This clarification has been added to the Methods section (Lines 238–240), and the terminology has been standardized throughout the Results and Discussion sections.　Furthermore, the indicators included in this model were originally designed to enable the identification of individuals at elevated risk of oral frailty in large-scale epidemiological and public health studies, where clinical oral examinations are often not feasible. Although previous studies have reported acceptable validity for self-reported oral health measures (Pitapat W, Garcia RI, Douglass CW, Joshipura KJ. Validation of self-reported oral health measures. J Public Health Dent. 2002 Spring;62(2):122-8. Doi: 10.1111/j.1752-7325.2002.tb03432.x. PMID: 11989207.), the outcome in the present study should be interpreted as a population-level risk indicator rather than a clinical diagnosis. This point has also been acknowledged as a limitation and has been added to the Limitations section (Lines 540–546).***

***Methods section (Lines 238–240): “In this study, oral frailty was estimated based on self-reported non-clinical indicators and is therefore distinct from a clinical diagnosis. Self-reported measures of dental and oral health status have been reported to show a certain degree of validity in previous studies.”***

***Limitations section (Lines 540–546): “First, oral frailty in this study was estimated using self-reported non-clinical indicators rather than a clinical diagnosis. Although previous studies have reported a certain degree of validity for self-reported measures of oral health status [35], the outcome used in this study represents a predicted risk of oral frailty at the population level rather than a clinically confirmed diagnosis. Therefore, some degree of misclassification may have occurred. The findings should thus be interpreted as reflecting relative differences in oral frailty risk rather than definitive clinical diagnoses.”***

3)

Third, while the SES measure (equivalent income) and social capital dimensions are well defined, the rationale for dichotomizing SES at 2 million yen could be clarified. Additionally, although the authors note adjustment for potential confounders, the selection criteria for covariates and whether any sensitivity analyses were conducted to test alternative specifications (STROBE Item 12e, 17) are not stated. Reporting on robustness checks would strengthen the internal validity of the findings.

***⇒ Thank you for your comment. Regarding the SES classification criteria, we set them with reference to the average equivalent disposable household income among households with individuals aged 65 years or older in Japan, as well as the distribution of equivalent household income in our study sample. According to the National Survey of Family Income and Expenditure, the average equivalent disposable household income for households with individuals aged 65 or older was 2.653 million yen in 2018 and 2.794 million yen in 2020. In this study, due to limitations in the JAGES survey items, some data required to calculate equivalent disposable income were unavailable. Therefore, we calculated equivalent household income and used it as the SES indicator. The Low-SES group was intended to reflect a low-income group below the national average, while the High-SES group reflects a middle-to-high-income group. The threshold of 2 million yen was adopted because it is below the national average for households with individuals aged 65 or older and, based on the distribution in our sample, allowed for a sufficient number of participants in each group. We have added this rationale for the binary SES classification in the manuscript (Lines 250-253).***

***(Lines 250-253): “These categories were defined with reference to the average equivalent disposable household income among households with individuals aged 65 years or older in Japan, as well as the distribution of equivalent household income in the study sample.”***

***⇒ In accordance with STROBE Items 12e and 17, we conducted a sensitivity analysis using the 2019 survey data. Relevant descriptions have been added to the Methods (Lines 324–329) and Results (Lines 466-476) sections.***

***Methods section (Lines 324–329): “To assess the robustness of the results, a sensitivity analysis was conducted using logistic regression with the 2019 survey data. According to the inclusion and exclusion criteria of the present study, the analytic sample consisted of 118,580 participants. The dependent variable was the presence or absence of oral frailty risk. Covariates included sex, age, education years, household composition, and number of chronic diseases. The independent variables were SES and health-related social capital.”***

***Results section (Lines 466-476)：”The sensitivity analysis revealed significant associations in both the low- and high-SES groups for community-level civic participation (low SES: OR = 0.249, 95% CI: 0.223–0.278; high SES: OR = 0.334, 95% CI: 0.301–0.372), reciprocity (low SES: OR = 0.427, 95% CI: 0.290–0.629; high SES: OR = 0.512, 95% CI: 0.349–0.751), and overall social capital (low SES: OR = 0.544, 95% CI: 0.508–0.583; high SES: OR = 0.616, 95% CI: 0.576–0.659), as well as for individual-level civic participation (low SES: OR = 0.864, 95% CI: 0.849–0.878; high SES: OR = 0.879, 95% CI: 0.866–0.893), social cohesion (low SES: OR = 0.925, 95% CI: 0.911–0.940; high SES: OR = 0.931, 95% CI: 0.916–0.947), reciprocity (low SES: OR = 0.843, 95% CI: 0.817–0.869; high SES: OR = 0.885, 95% CI: 0.851–0.920), and overall social capital (low SES: OR = 0.910, 95% CI: 0.901–0.919; high SES: OR = 0.917, 95% CI: 0.908–0.926).”***

4)

Fourth, the stratified analysis by SES adds value, but subgroup sizes and event counts in each group should be reported directly to allow readers to assess precision. Furthermore, interpretation of effect sizes should be presented cautiously, especially given the relatively small differences in mean scores and odds ratios near 1.0 (e.g., OR 0.947, 95% CI: 0.932–0.963 for individual-level social capital).

***⇒ Thank you for this helpful suggestion. The number of participants and oral frailty risk events in each SES subgroup were already reported in Table 1 (The risk of oral frailty and characteristics of the participants). In this table, the event corresponds to participants classified as having predicted oral frailty risk (p ≥ 0.1824) according to the Oral Frailty Predictive Model developed by Yamamoto et al. (2022). To make this information clearer for readers, we have added a brief clarification in the Results section (Lines 362–365) explicitly referring to the subgroup sample sizes and event counts presented in Table 1. We appreciate the reviewer’s comment, which helped us improve the transparency of the reporting.***

***(Lines 362–365): “Among those with oral frailty risk, 24,587 individuals (78.4%) were from the total sample (n = 31,378), 8,796 (75.4%) from the low-SES group (n = 11,662), and 15,791 (80.1%) from the high-SES group (n = 19,716).”***

5)

Fifth, the discussion is generally well reasoned, and limitations are acknowledged. However, some conclusions remain slightly overextended in terms of policy recommendations. For example, while the study supports associations between social capital and oral frailty risk, statements that interventions “may contribute to the sustainability of social security systems” or that social capital “reduces risk” could be rephrased more conservatively, given the observational nature of the data.

***⇒ Thank you for this valuable comment. We agree that some statements in the original manuscript could be interpreted as overly strong given the observational nature of the study.　Accordingly, we revised the relevant sentences in the Discussion section (Lines 576-586) to present the implications more cautiously. In particular, statements suggesting that social capital “reduces oral frailty risk” or “may contribute to the sustainability of social security systems” have been rephrased to emphasize that the findings indicate associations rather than causal effects, and that policy implications should be interpreted with appropriate caution.***

***(Lines 576-586): “Overall, these findings provide a potential basis for considering cost-effective interventions aimed at enhancing social capital and highlight potential associations with the incidence of oral frailty risk, irrespective of SES. At the individual level, the observed associations suggest that higher social capital may be linked to lower oral frailty risk among low-income individuals, which could have implications for healthy life expectancy. At the community level, higher social capital may be associated with aspects of community development. From a societal perspective, these associations could inform discussions on strategies to support the sustainability of social security systems; however, given the observational nature of this study, any policy implications should be interpreted with appropriate caution. Future research should identify the characteristics of communities and individuals with high social capital and develop, implement, and evaluate interventions to further investigate these relationships.”***

6)

Sixth, the manuscript does not report whether any efforts were made to address potential bias, including unmeasured confounding or selection bias due to loss to follow-up (STROBE Item 9, 12d). While the exclusion criteria are thorough, the potential for differential loss should be discussed in more detail.

***⇒ Thank you for highlighting this important issue. While we attempted to reduce confounding by adjusting for a range of demographic and health-related covariates, we acknowledge that residual confounding and selection bias cannot be completely ruled out.　To address this concern, we have added a more detailed discussion in the Limitations section (Lines 546-550). Specifically, we now note that unmeasured confounding may remain despite multivariable adjustment, and that selection bias due to loss to follow-up may have influenced the observed associations if participants lost to follow-up differed systematically from those who remained in the study.***

***(Lines 546-550): “Second, while we attempted to reduce confounding by adjusting for a range of demographic and health-related covariates, we acknowledge that residual confounding cannot be completely ruled out. In addition, selection bias due to loss to follow-up may have influenced the observed associations if participants lost to follow-up differed systematically from those who remained in the study.”***

7)

Seventh, the statistical methods are clearly described, and the models are adjusted for relevant demographic and health-related covariates. Odds ratios and confidence intervals are presented appropriately. However, as noted above, the paper would benefit from sensitivity analyses—such as alternative SES thresholds, interaction terms, or models excluding certain predictors—to assess robustness.

***⇒ We sincerely appreciate the reviewer’s constructive suggestions. We also agree that conducting additional analyses is useful for assessing the robustness of the study findings. In response to the editorial suggestions, we performed supplementary analyses to further examine the relationships hypothesized in this study. Specifically, to evaluate the robustness of the logistic regression results, we conducted a sensitivity analysis using the 2019 survey data. Additionally, to explore the potential mediating role of social capital in the association between socioeconomic status (SES) and oral frailty risk, we performed mediation analysis using structural equation modeling (SEM). These approaches allowed us not only to confirm the robustness of the logistic regression findings but also to examine the underlying pathways connecting SES, social capital, and oral frailty risk within a unified analytical framework. The results of these analyses were generally consistent with the main findings and further supported the conceptual model proposed in this study. Accordingly, we have added a description of these analyses to the Methods section (Lines 324–329, 329-333) and reported the corresponding results in the Results section (Lines 422–430, 444-452, 466-476, Figure 2-9). We appreciate the reviewer’s comment, which has contributed to enhancing the transparency of our analyses and the interpretability of the findings.***

***Methods section (Lines 324–329)：”To assess the robustness of the results, a sensitivity analysis was conducted using logistic regression with the 2019 survey data. According to the inclusion and exclusion criteria of the present study, the analytic sample consisted of 118,580 participants. The dependent variable was the presence or absence of oral frailty risk. Covariates included sex, age, education years, household composition, and number of chronic diseases. The independent variables were SES and health-related social capital.”***

***(Lines 329-333): “For the social capital variables that showed significant associations with oral frailty risk in the low-SES group in the logistic regression analyses, structural equation modeling (SEM) was conducted for both the low-SES and high-SES groups to analyze the mediating structure. Model fit was evaluated using the chi-square test, GFI, CFI, and RMSEA.”***

***Results section (Lines 422–430): “Fig 2–5 present the results of SEM examining the mediating role of social capital indicators that showed significant associations in the low-SES group. Fig 2 represents the mediating structure of Community-level Civic participation (χ²(4) = 109.006, GFI = .996, CFI = .854, RMSEA = .047), Fig 3 represents the mediating structure of Individual-level Civic participation (χ²(1) = 2.240, GFI = 1.000, CFI = .998, RMSEA = .010), Fig 4 represents the mediating structure of Individual-level Social cohesion (χ²(1)*** ***= 1.721, GFI = 1.000, CFI = .998, RMSEA = .008), and Fig 5 represents the mediating structure of Individual-level Overall Social Capital (χ²(1) = .128, GFI = 1.000, CFI = 1.000, RMSEA = .000). These findings are consistent with the associations observed in the logistic regression analyses.”***

***(Lines 444-452): “Fig 6–9 present the results of SEM in the high-SES group, corresponding to the Social capital indicators examined in the low-SES group. Fig 6 represents the mediating structure of Community-level Civic participation (χ²(1) = 3.320, GFI = 1.000, CFI = .997, RMSEA = .011), Fig 7 represents the mediating structure of Individual-level Civic participation (χ²(1) = 9.122, GFI = 1.000, CFI = .988, RMSEA = .020), Fig 8 represents the mediating structure of Individual-level Social cohesion (χ²(1) = .151, GFI = 1.000, CFI = 1.000, RMSEA = .000), and Fig 9 represents the mediating structure of Individual-level Overall Social Capital (χ²(1) = 3.441, GFI = 1.000, CFI = .996, RMSEA = .011). These findings are consistent with the associations observed in the logistic regression analyses.”***

***(Lines 466-476): “The sensitivity analysis revealed significant associations in both the low- and high-SES groups for community-level civic participation (low SES: OR = 0.249, 95% CI: 0.223–0.278; high SES: OR = 0.334, 95% CI: 0.301–0.372), reciprocity (low SES: OR = 0.427, 95% CI: 0.290–0.629; high SES: OR = 0.512, 95% CI: 0.349–0.751), and overall social capital (low SES: OR = 0.544, 95% CI: 0.508–0.583; high SES: OR = 0.616, 95% CI: 0.576–0.659), as well as for individual-level civic participation (low SES: OR = 0.864, 95% CI: 0.849–0.878; high SES: OR = 0.879, 95% CI: 0.866–0.893), social cohesion (low SES: OR = 0.925, 95% CI: 0.911–0.940; high SES: OR = 0.931, 95% CI: 0.916–0.947), reciprocity (low SES: OR = 0.843, 95% CI: 0.817–0.869; high SES: OR = 0.885, 95% CI: 0.851–0.920), and overall social capital (low SES: OR = 0.910, 95% CI: 0.901–0.919; high SES: OR = 0.917, 95% CI: 0.908–0.926).”***

Overall, this is a rigorous and policy-relevant study that offers valuable insight into the intersection of SES, social capital, and aging-related oral health. A few clarifications on methodological decisions and greater transparency in reporting would enhance the manuscript further. I hope the authors find these suggestions helpful.

***⇒ We sincerely appreciate the reviewer’s positive and encouraging evaluation of our work. Your thoughtful suggestions have helped us improve the clarity, transparency, and methodological rigor of the manuscript. We have carefully revised the manuscript in accordance with your comments and believe that these revisions have strengthened the presentation and interpretation of our findings.***

***Thank you again for your valuable feedback.***

**To Reviewer 2**

I thank the authors for submitting this manuscript addressing an important and timely topic. The investigation of social capital as a potential buffer against oral frailty risk among older adults with lower socioeconomic status represents a meaningful contribution to the gerontological and public health literature. The use of longitudinal data from a large, well-established cohort study is commendable, and the research question has clear implications for healthy aging policies.

While the manuscript addresses a relevant question with appropriate data, several conceptual and methodological issues require attention before the work can be considered for publication. Most critically, there is a fundamental inconsistency between the stated aim (examining "mediating effects") and the analytical approach employed (stratification by SES). Stratification examines whether the association between social capital and oral frailty differs across SES strata and this is effect modification or moderation, not mediation. Throughout the manuscript, there is no mention of formal mediation analysis techniques (e.g., causal mediation analysis, structural equation modeling). I strongly recommend that the authors either (a) revise the title, aims, and framing to accurately reflect a moderation analysis, or (b) conduct and report proper mediation analyses if the original research question is indeed about mediation pathways.

Additionally, the authors describe their work as a "multilevel approach," which may mislead readers familiar with multilevel (hierarchical) regression models. The current analysis aggregates community-level variables and includes them as individual-level predictors in standard logistic regression, which does not account for the nested data structure (individuals within municipalities). A true multilevel model would better accommodate community-level effects and the clustering of participants. If such modeling was not feasible, this should be acknowledged as a limitation, with recommendations for future research.

Below, I present section-specific questions and some comments aimed at helping improve the reporting of the investigation.

***⇒ Thank you very much for your thoughtful and constructive comments. We sincerely appreciate your careful reading of our manuscript and your valuable suggestions, which helped us improve both the conceptual clarity and methodological transparency of the study.***

***In response to your important comment regarding the inconsistency between the stated aim (mediation) and the analytical approach originally presented, we conducted an additional mediation analysis using structural equation modeling (SEM) to formally examine the potential mediating role of social capital in the association between socioeconomic status (SES) and oral frailty risk. The results of this mediation analysis have now been added to the manuscript and are presented in the Methods (Lines 329-333) and Results section (Lines 422–430, 444-452) and illustrated in Figure 2-9. We believe that this additional analysis clarifies the analytical framework and strengthens the interpretation of the relationships examined in this study. We are grateful for your suggestion, which substantially improved the methodological rigor of our work.***

***Methods section (Lines 329-333): “For the social capital variables that showed significant associations with oral frailty risk in the low-SES group in the logistic regression analyses, structural equation modeling (SEM) was conducted for both the low-SES and high-SES groups to analyze the mediating structure. Model fit was evaluated using the chi-square test, GFI, CFI, and RMSEA.”***

***Results section (Lines 422–430): “Fig 2–5 present the results of SEM examining the mediating role of social capital indicators that showed significant associations in the low-SES group. Fig 2 represents the mediating structure of Community-level Civic participation (χ²(4) = 109.006, GFI = .996, CFI = .854, RMSEA = .047), Fig 3 represents the mediating structure of Individual-level Civic participation (χ²(1) = 2.240, GFI = 1.000, CFI = .998, RMSEA = .010), Fig 4 represents the mediating structure of Individual-level Social cohesion (χ²(1) = 1.721, GFI = 1.000, CFI = .998, RMSEA = .008), and Fig 5 represents the mediating structure of Individual-level Overall Social Capital (χ²(1) = .128, GFI = 1.000, CFI = 1.000, RMSEA = .000). These findings are consistent with the associations observed in the logistic regression analyses.”***

***(Lines 444-452): “Fig 6–9 present the results of SEM in the high-SES group, corresponding to the Social capital indicators examined in the low-SES group. Fig 6 represents the mediating structure of Community-level Civic participation (χ²(1) = 3.320, GFI = 1.000, CFI = .997, RMSEA = .011), Fig 7 represents the mediating structure of Individual-level Civic participation (χ²(1) = 9.122, GFI = 1.000, CFI = .988, RMSEA = .020), Fig 8 represents the mediating structure of Individual-level Social cohesion (χ²(1) = .151, GFI = 1.000, CFI = 1.000, RMSEA = .000), and Fig 9 represents the mediating structure of Individual-level Overall Social Capital (χ²(1) = 3.441, GFI = 1.000, CFI = .996, RMSEA = .011). These findings are consistent with the associations observed in the logistic regression analyses.”***

***⇒ In addition, we appreciate your comment regarding the use of the term “multilevel approach.” As you correctly noted, our analysis did not employ a hierarchical multilevel regression model that explicitly accounts for the nested data structure of individuals within municipalities. To avoid potential misunderstanding among readers familiar with multilevel modeling, we have revised the terminology throughout the manuscript and removed the expression “multilevel approach,” replacing it with wording that more accurately reflects our analytical strategy.***

***We thank the reviewer again for these insightful comments, which helped us improve the clarity, accuracy, and overall quality of the manuscript.***

***(Lines 177-183): “This study holds both theoretical and practical importance: theoretically, it advances understanding of the SES–oral frailty risk pathway by considering social capital at both the community and individual levels as potential mediators; practically, it identifies modifiable factors at both the community and individual levels that can inform targeted interventions for older adults with low SES, thereby contributing to health equity within the framework of the Social Determinants of Health model.”***

ABSTRACT

1)

The phrasing "longitudinal data from the Japan Gerontological Evaluation Study (JAGES) collected in 2019 and 2022" is imprecise. "In 2019 and 2022" denotes two discrete time points, not a range. Consider revising to "collected at baseline (2019) and follow-up (2022)" or similar phrasing that accurately reflects the two-wave design.

***⇒ Thank you very much for your valuable comment. You are correct that the original wording could have caused misunderstanding. To more accurately reflect the two-wave study design, we have revised the text in the manuscript as suggested (Lines 33-34).***

***(Lines 33-34): “We analyzed longitudinal data from the Japan Gerontological Evaluation Study (JAGES) collected at baseline (2019) and follow-up (2022)”***

INTRODUCTION

2)

The manuscript would benefit from a clear definition of "healthy life expectancy," briefly characterizing what is understood as health during this life stage, particularly in the Japanese societal context. Does it mean maintaining the integrity of physiological and psychological capacities? Is it related to individual autonomy and social integration? A brief clarification would anchor the reader's understanding.

***⇒ Thank you for this helpful suggestion. We agree that a clearer explanation of the concept of healthy life expectancy would help readers better understand the context of this study. In response, we have added a brief clarification in the Introduction (Lines 62–70).***

***In the revised manuscript, healthy life expectancy is described as the period during which individuals can maintain functional health and independence in daily life, including the preservation of physiological and psychological capacities, as well as the ability to participate in social activities. We also briefly explain that, in Japan, the extension of healthy life expectancy has been a central public health policy goal in response to rapid population aging. We appreciate the reviewer’s suggestion, which helped improve the conceptual clarity of the manuscript.***

***(Lines 62-70): According to the World Health Organization (WHO), healthy life expectancy is defined as the average number of years that a person can expect to live in "full health", if he or she were to pass through the remaining years of life exposed to the sex- and age-specific mortality and morbidity prevailing at the time, for a specific year, in a given country, territory, or geographic area. In other words, it represents the period during which an individual can maintain functional health and independence in daily life, encompassing not only the preservation of physical and psychological functions but also the ability to participate in social activities. In Japan, extending healthy life expectancy has become a central public health goal in response to the rapidly aging population [3].***

3)

I am not sure the argument that health inequalities "widen with age and become more pronounced later in life" is clearly stated. Why would that be? The mechanism or evidence supporting this claim should be briefly articulated.

***⇒ Thank you for this important comment. We agree that the mechanisms underlying the widening of health inequalities in later life should be more clearly articulated. In response, we have revised the relevant sentence in the Introduction (Lines 76-85) to briefly explain the potential mechanisms.***

***Specifically, we now note that socioeconomic inequalities in health may accumulate over the life course through differences in material resources, health behaviors, and access to supportive social environments. These inequalities often begin early in life and tend to widen with age, becoming particularly pronounced in the later stages of life. These cumulative disadvantages may lead to greater disparities in functional health outcomes in older age. By clarifying this life-course perspective, we aim to provide a clearer rationale for examining socioeconomic differences in oral frailty risk among older adults.***

***(Lines 76-85): Functional health—including respiratory capacity, muscular strength, and cardiovascular performance—reaches its peak in early adulthood and gradually declines with advancing age. The rate of this decline may vary depending on individual characteristics, living conditions, and behavioral factors [5]. In this sense, socioeconomic disparities in health may accumulate across the life course through differences in material resources, health behaviors, and access to supportive social environments. Such cumulative disadvantages may further widen inequalities in functional health outcomes in later life, a process often described as cumulative advantage (or disadvantage). Previous research suggests that cumulative advantage/disadvantage can be defined as the systemic tendency for interindividual divergence in a given characteristic (e.g., money, health, or status) with the passage of time [6].***

4)

Is the problem really "identifying effective and equitable strategies to reduce health disparities regardless of SES," or is it more precisely to account for the influences of SES on said health disparities? The current framing seems to understate the centrality of SES as a structural determinant.

***⇒ Thank you for this insightful comment. We agree that socioeconomic status (SES) should be more clearly framed as a structural determinant of health disparities, rather than being treated merely as a factor to be controlled for.***

***Accordingly, we have revised the relevant description in the Introduction (Lines 89–91) to emphasize that SES plays a fundamental role in shaping health inequalities among older adults. The revised text now highlights the importance of understanding how social determinants, including SES, influence oral health outcomes and related functional decline. This clarification better reflects the conceptual framework of the study and strengthens the rationale for examining SES-stratified associations.***

***(Lines 89-91): SES is a structural determinant of health that is not easily modified and plays a fundamental role in shaping health inequalities [9].***

5)

How does access to oral health services impact oral frailty in the context of Japan? Do older adults have universal access to dental care? Is SES relevant because only more privileged individuals are granted access to oral health care, or are there other pathways? I believe this aspect of the Japanese healthcare system should be clarified, as it may significantly blur the interpretation of SES effects.

***⇒ Thank you for raising this important point. We agree that providing context about the Japanese healthcare system and access to dental services would help readers interpret the role of SES in oral health outcomes.***

***In response, we have added a brief explanation in the Introduction (Lines 144-149) describing the characteristics of dental care access in Japan. Specifically, we note that Japan has a universal health insurance system that covers most dental treatments, which allows older adults to access oral healthcare services with relatively low out-of-pocket costs. Therefore, socioeconomic differences in oral frailty risk may not be explained solely by differential access to dental services. Instead, SES-related disparities may operate through other pathways, such as differences in health behaviors, social participation, and social resources, including social capital.***

***(Lines 144-149): “Japan has a universal health insurance system that covers most dental treatments, allowing older adults to access oral healthcare services with relatively low out-of-pocket costs. Consequently, socioeconomic disparities in oral frailty risk may not be solely attributable to differences in access to dental care, and such disparities may arise through alternative pathways, including variations in other social resources.”***

METHODS

6)

Which 76 municipalities were included? Out of how many total municipalities in Japan? Providing this context would help readers assess the generalizability of findings.

***⇒Thank you for your valuable comment. The 76 municipalities refer to those that participated in the 2022 survey. Among these, 49 municipalities took part in both the 2019 and 2022 surveys.　In Japan, there are a total of 1,718 municipalities across all 47 prefectures, spanning eight major regions: Hokkaido, Tohoku, Kanto, Chubu, Kinki, Chugoku, Shikoku, and Kyushu. The 49 municipalities included in our analysis were drawn from across these regions nationwide.　We have added this information to the manuscript to provide context for assessing the generalizability of our findings. (Lines 199-201).***

***(Lines 199-201): “Participants were recruited from 49 municipalities that participated in both the 2019 and 2022 surveys, out of a total of 1,718 municipalities across all 47 prefectures in Japan.***

7)

Was a baseline sample size estimated a priori? If so, please report the parameters used.

***⇒ Because the present study was a secondary analysis of an existing dataset, an a priori sample size calculation was not conducted. However, a post hoc power analysis based on the observed incidence rates (24.6% vs. 19.9%) and the total sample size (Low-SES = 11,662； Higi-SES= 19,716) indicated that the statistical power exceeded 90% at a significance level of 0.05. Therefore, the available sample size was more than sufficient to detect the observed association.***

8)

How were participants randomized in municipalities with over 5,000 residents? The sampling procedure should be described in sufficient detail for replication.

***⇒ Thank you for this important comment. We agree that a clearer description of the sampling procedure is necessary to ensure reproducibility. We have therefore revised the Methods section (Lines 202-204) to provide additional details:***

***(Lines 202–204): “in municipalities with more than 5,000 eligible residents, participants were selected using stratified random sampling based on the municipal resident registry and social insurance records related to functional independence certification.”***

9)

The exclusion criteria appear very strict. While I understand the rationale for ensuring a functionally independent baseline sample, this should be acknowledged when discussing generalizability.

***⇒ Thank you for this insightful comment. As the reviewer noted, the exclusion criteria in this study were relatively strict to ensure a cohort of functionally independent older adults at baseline. However, we acknowledge that this approach may affect the external validity of the findings. Accordingly, we have added a statement in the Limitation (Lines 550-554) noting that the results may primarily apply to relatively healthy and functionally independent older adults, and we have described this point as a limitation of the study.***

***(Lines 550-554): “Third, the exclusion criteria in this study were relatively strict in order to ensure a cohort of functionally independent older adults at baseline. Consequently, the findings may primarily reflect outcomes among relatively healthy and functionally independent individuals, and caution is warranted when generalizing the results to the broader population of older adults.”***

10)

①What is the exact reasoning behind the differential weighting of community-level indicators of social capital? As currently presented, the different weights seem arbitrary. In the referenced Saito et al. original scale, there is no such weighting suggestion; rather, factor scores are estimated. A clear justification should be presented for the weighting scheme used. ②Furthermore, in their investigation, Saito et al. use multilevel regression to model exposure and outcomes. Was this not possible in the current investigation? A multilevel model would better account for common community-level variation in social capital and the clustering of individuals within municipalities.

***⇒ We appreciate the reviewer’s insightful comment.
① The weighting scheme used in this study was derived from the factor loadings reported in the original scale development by Masashige Saito and colleagues in the development of the JAGES community-level social capital indicators（available at the JAGES project website, https://www.jages.net/library/social-capital/?action=common_download_main&upload_id=12095）. To clarify this point, we have revised the Methods section to explicitly describe the rationale and source of the weighting scheme and to cite the relevant methodological documentation from the JAGES project. 　The revised sentence in the manuscript now reads as follows:***

***(Lines 258–262): “Community-level social capital indicators were calculated using weighted composite scores based on the factor loadings reported in the original JAGES social capital scale development study. The weighting procedure followed the calculation formula provided in the JAGES methodological documentation [37], which reflects the relative contribution of each indicator to the latent social capital construct.”***

***② We fully agree that multilevel modeling is an appropriate approach for accounting for clustering of individuals within communities and for simultaneously examining individual- and community-level effects. Indeed, the original study by Masashige Saito and colleagues employed multilevel regression models for this purpose.　In the present study, however, our primary analytical focus was to examine the association between individual characteristics and the provision of social support while incorporating community-level social capital indicators as contextual variables. Given the analytical objectives and the structure of the available dataset, we employed conventional regression models with municipality-level aggregated indicators.　Nevertheless, we acknowledge that multilevel modeling could provide a more rigorous framework for accounting for within-municipality clustering and contextual variation. We have therefore added a statement in the Discussion section acknowledging this methodological consideration and identifying the use of multilevel models as an important direction for future research.　The manuscript has been revised accordingly as follows:***

***(Lines 554–558): “Fourth, although community-level social capital indicators were included as contextual variables in the present analyses, the use of conventional regression models may not fully account for clustering of individuals within municipalities. Future studies employing multilevel modeling would allow for a more precise examination of both individual- and community-level contributions to social support provision.”***

11)

Is it possible to specify which chronic diseases were considered and how they were modeled in the regression (e.g., score range, categorization)?

***⇒ We appreciate the reviewer’s request for clarification regarding the chronic disease variable. In this study, chronic diseases were assessed using self-reported physician diagnoses for the following conditions commonly included in the Japan Gerontological Evaluation Study (JAGES): hypertension, stroke, heart disease, diabetes, hyperlipidemia, respiratory diseases, gastrointestinal/liver/gallbladder diseases, kidney/prostate diseases, musculoskeletal disorders, trauma, cancer, hematologic/immune disorders, depression, dementia, Parkinson’s disease, eye diseases, ear diseases. These conditions were selected because they represent major chronic diseases affecting older adults in Japan and are widely used as health status indicators in epidemiological studies based on JAGES data.　For the regression analyses, the presence of chronic diseases was operationalized as a categorical variable. To clarify this point, we have revised the Methods section to explicitly describe the chronic disease variables and their operationalization in the statistical analyses (Lines 299-305).***

***(Lines 299-305): “The number of chronic conditions was calculated by dichotomizing the presence or absence of 17 chronic diseases (hypertension, stroke, heart disease, diabetes, hyperlipidemia, respiratory diseases, gastrointestinal/liver/gallbladder diseases, kidney/prostate diseases, musculoskeletal disorders, trauma, cancer, hematologic/immune disorders, depression, dementia, Parkinson’s disease, eye diseases, and ear diseases) as categorical variables and summing them. The score ranged from 0 to 17.”***

12)

It would be helpful if the authors could better describe attrition and missing data at baseline and follow-up, particularly for the 40,000 participants excluded from analysis. Do excluded participants differ systematically from those retained? Of those, how many presented with oral frailty risk at baseline?

***⇒ We thank the reviewer for highlighting the importance of providing greater transparency regarding participant attrition and missing data. Participants were excluded from the final analytic sample primarily due to missing responses on key variables, including oral frailty indicators, equivalent household income, or covariates required for the regression analyses. In addition, some participants were excluded because they did not respond to the follow-up survey. To address the reviewer’s concern, we conducted additional descriptive analyses comparing the characteristics of participants included in the final analysis with those who were excluded. The results indicate that excluded participants tended to be older and had slightly poorer health status than those retained in the analytic sample. We have added information on the number of excluded participants who presented with oral frailty risk at baseline (Lines 355-356).***

***(Lines 355-356): “At baseline, 37,695 participants were identified as having a risk of oral frailty.”***

13)

Could the authors provide better context regarding the equivalent household income variable? Why was the 2 million yen threshold chosen for segregating SES groups? Is this based on minimum wage, an indicator of poverty, or a similar underprivileged situation in Japan? While this may be clear to those familiar with the Japanese economy, for a broader international readership it would be helpful to have additional explanation given that this is the main exposure variable.

***⇒ Thank you for your comment. Regarding the SES classification criteria, we set them with reference to the average equivalent disposable household income among households with individuals aged 65 years or older in Japan, as well as the distribution of equivalent household income in our study sample. According to the National Survey of Family Income and Expenditure, the average equivalent disposable household income for households with individuals aged 65 or older was 2.653 million yen in 2018 and 2.794 million yen in 2020. In this study, due to limitations in the JAGES survey items, some data required to calculate equivalent disposable income were unavailable. Therefore, we calculated equivalent household income and used it as the SES indicator. The Low-SES group was intended to reflect a low-income group below the national average, while the High-SES group reflects a middle-to-high-income group. The threshold of 2 million yen was adopted because it is below the national average for households with individuals aged 65 or older and, based on the distribution in our sample, allowed for a sufficient number of participants in each group. We have added this rationale for the binary SES classification in the manuscript (Lines 250-253).***

***(Lines 250-253): “These categories were defined with reference to the average equivalent disposable household income among households with individuals aged 65 years or older in Japan, as well as the distribution of equivalent household income in the study sample.”***

RESULT

14)

Table 2 could be consolidated as a first or final row in Table 1, rather than presented as a separate table. Additionally, in terms of descriptive potential, an initial table might be more informative if structured by the outcome variable (oral frailty risk) rather than by SES level.

***⇒ Thank you for your comment. Based on your suggestion, we considered that combining Table 2 with Table 1 would improve clarity. Therefore, we have integrated Table 2 into Table 1 and revised the table title to “The risk of oral frailty and characteristics of the participants.” (Line 368)***

15)

I would recommend reconsidering the presentation of oral frailty incidence by person-years (lines 314–315), as this metric is not explored elsewhere in the manuscript and may confuse readers given the discrete two-wave design.

***⇒ Thank you for your comment. In this study, we initially calculated incidence using the person-year method as a supplementary measure to facilitate comparison with previous epidemiological studies. However, as you rightly pointed out, our study employed a two-timepoint design, and thus presenting person-year incidence was not strictly necessary. Accordingly, we have removed the description of incidence calculated using the person-year method from the main text.***

16)

The mean values for community-level indicators are nearly identical across SES groups (indeed, identical for reciprocity), with statistical significance likely driven by sample size rather than meaningful differences. This limited between-group variation in community-level social capital may explain the null findings for community-level moderation effects reported later, a point worth acknowledging in the discussion as a potential limitation of the analytic strategy rather than evidence that community-level social capital is unimportant.

***⇒ We agree with the reviewer that the minimal differences in community-level social capital indicators across SES groups may limit the ability to detect moderation effects at the community level. To address this concern, we have added a statement in the Discussion acknowledging that the limited between-group variation in community-level social capital constitutes a methodological limitation. The revised text in the Discussion now reads:***

***(Lines 558-563): “Fifth, it should be noted that the mean values of community-level social capital indicators were very similar across SES groups, with reciprocity showing virtually no variation. As a result, the lack of observed moderation effects at the community level may reflect the limited between-group variation rather than indicating that community-level social capital is unimportant. This limitation of the analytic strategy should be considered when interpreting the findings.”***

DISCUSSION

17)

I believe it would be valuable to add a sentence or two in the discussion regarding the dissipation of sex effects on oral frailty in the high-SES group across all three models. Why does being a woman appear protective among low-SES participants but not among high-SES participants? This pattern warrants interpretation.

***⇒ We thank the reviewer for this insightful comment. The observed attenuation of the protective effect of female sex on oral frailty in the high-SES group warrants interpretation.　In our analyses, being female was associated with lower oral frailty risk among low-SES participants, whereas this effect was diminished and non-significant among high-SES participants. One possible explanation is that women in lower-SES groups may engage more actively in health-promoting oral behaviors, such as maintaining oral hygiene or seeking dental care, which may confer measurable protective effects when resources are otherwise limited. In high-SES groups, access to healthcare and preventive resources is generally more equitable between sexes, potentially reducing observable sex differences.　However, these explanations are hypothetical. We have added the following sentences to the Discussion to reflect this interpretation and highlight directions for future research:***

***(Lines 492-498): “Interestingly, the protective association of female sex with oral frailty risk was observed primarily among low-SES participants but was attenuated among high-SES participants. This pattern may reflect differences in health behaviors, with women in lower-SES groups potentially engaging more actively in oral health-promoting behaviors when resources are limited. In higher-SES groups, more equitable access to healthcare and preventive services may reduce sex differences in oral frailty risk. Importantly, further research is needed to directly examine the mechanisms underlying the observed sex × SES interaction.”***
