# Supplementary material for: Associations between health-related social capital and oral frailty risk among older adults: A stratified analysis by socioeconomic status in the JAGES longitudinal study
Source: PLoS One. 2026 Jul 24;21(7):e0353850. doi: 10.1371/journal.pone.0353850 (PMC13399358; doi:10.1371/journal.pone.0353850)
Supplement: Attachment — Submitted filename: Response to Reviewers.docx [file pone.0353850.s004.docx]

To Reviewer 2

**Comment 1　MEDIATION ANALYSIS IS INCONSISTENT WITH THE MANUSCRIPT:**

Based on Figure 2, for instance, the path diagram depicts SES as the exposure, with direct paths from SES to education, household composition, sex, and oral frailty risk, and an indirect path from SES through community-level civic participation to oral frailty risk. This is, structurally, a mediation model in which SES is the exposure. However, the figure title ("Mediating structure of Community-level Civic participation in Low-SES for Oral frailty risk") and the methods text ("SEM was conducted for both the low-SES and high-SES groups to analyze the mediating structure") describe stratified estimation within SES subgroups. These are incompatible: SES cannot simultaneously be the exposure in a fitted path model and the variable used to split the sample into two separate models. If the mediation model in Figure 2 was estimated within the low-SES subsample, the SES → civic participation path cannot be meaningfully estimated because within-subsample variance in SES is restricted by the stratification itself. Conversely, if SES is a functioning exposure variable in the model, then the "in Low-SES" labeling is inaccurate. The authors should clarify exactly what was estimated.

Beyond this internal inconsistency, the model specification in Figure 2 raises concerns even when taken on its own terms. The paths SES → sex and SES → education cannot be interpreted causally, since sex is determined at birth and educational attainment is typically completed decades before SES measurement in this cohort. This suggests the arrows are being used as statistical regression paths rather than directed causal relationships, which is required by mediation analysis for the indirect effect to have meaning. Additionally, modeling covariates (education, household composition, sex) as outcomes of the exposure rather than as exogenous adjustment variables changes the meaning of the direct and indirect effects in ways the manuscript does not address. Covariates should typically be modeled as exogenous predictors of the mediator and outcome (Valeri & VanderWeele, 2013, Psychological Methods, 18(2), 137–150).

Finally, no decomposition of total, direct, and indirect effects is reported, which is the purpose of a mediation analysis. The authors' statement that the SEM findings "are consistent with the associations observed in the logistic regression analyses" suggests the SEM is being used descriptively to recover the regression estimate rather than to quantify mediation pathways.

Two further technical issues warrant clarification. First, oral frailty risk is a binary outcome, but the estimator used in Amos is not specified; default maximum likelihood estimation with a binary outcome produces somewhat biased estimates. Second, the decision to fit SEM only for social capital variables significant in the low-SES logistic regression is a post-hoc selection step that compromises interpretation of the corresponding high-SES models, which are estimated conditionally on results from the other stratum.　I recommend the authors take one of two paths. Either

(a) fit a unified mediation model with SES as exposure on the full sample and report indirect, direct, and total effect decomposition, or

(b) remove the mediation framing entirely, revise the title, aims, and discussion to present this as a moderation/effect modification study, and report the stratified analyses transparently as such.

***Response 1：***

*We sincerely thank the reviewer for the thorough and insightful evaluation of our manuscript and for the constructive comments regarding the inconsistency between the mediation framework and the analyses presented. We are also grateful for the additional clarification provided in the subsequent review. After carefully revisiting the reviewer’s comments, we recognized that our previous revision did not fully address the conceptual concerns regarding the mediation framework. Upon reconsideration, we fully agree that there were conceptual and methodological inconsistencies in framing the study as a mediation analysis. In particular, we agree that SES cannot simultaneously function as an exposure variable within a mediation model and as the variable used to stratify the study population. We also acknowledge that the SEM presented in the previous version did not provide sufficient evidence to support a mediation interpretation.*

*Accordingly, following the reviewer’s recommendation (b), we substantially restructured the manuscript. Specifically, we completely removed the mediation framework and revised the Title, Abstract, Introduction, Study Aims, Methods, Results, and Discussion sections (lines 1–3, 25–29, 36-39, 46-51, 54-57, 118-123, 130-136, 148–173, 236–250, 327–334, 391-394, 404-407, 415-418, 427-430, 453-459, 446–491) to present the study as an investigation of moderation/effect modification. Consistent with this conceptual revision, all SEM analyses, figures, and related descriptions have been removed from the manuscript. Furthermore, in accordance with the reviewer’s recommendation to “report the stratified analyses transparently as such,” we have explicitly reclassified the previously conducted analyses in the low-SES and high-SES groups as stratified analyses rather than mediation analyses. In addition, we have reframed the study to examine whether the association between SES and incident oral frailty differs according to levels of social capital.*

*To formally assess effect modification, we additionally conducted logistic regression analyses using the full study population. Specifically, we fitted models including SES, social capital (SC), an SES × SC interaction term, and relevant covariates (Frailty = SES + SC + SES × SC + covariates). The results showed that the interaction between SES and individual-level civic participation was statistically significant, indicating that the association between SES and incident oral frailty differed according to the level of individual-level civic participation. These findings support the effect-modification hypothesis and are consistent with the results of the stratified analyses. Importantly, this revised analytical framework avoids the conceptual inconsistencies associated with the previous mediation framework and more appropriately addresses the reviewer’s concern regarding the interpretation of the relationship between SES, social capital, and oral frailty.*

*The results of these analyses have been added to Tables 3–6, and lines 391-394, 404-407, 415-418, 427-430, and 453-459 have been revised accordingly.*

**Comment 2　MULTILEVEL MODELLING:**

The removal of "multilevel approach" from the manuscript is appropriate, and the added limitation at Lines 554–558 is a reasonable compromise. However, the rationale provided in the response letter is inconsistent with the study design. The authors state that "our primary analytical focus was to examine the association between individual characteristics and the provision of social support." Social support provision is not, as I understand it, the outcome of this study; oral frailty risk is. This phrasing suggests either a drafting error or that this justification was adapted from another context, and I would ask the authors to clarify.

More substantively, the authors note that Saito et al. employed multilevel regression with the JAGES community-level social capital indicators, which implies that the data structure does, in fact, support multilevel modeling. The constraint is therefore analytical choice rather than data availability. Thys, justification as currently written is not coherent and should be revised.

***Response 2：***

*We appreciate the reviewer’s careful reading of both the manuscript and our response letter. As the reviewer correctly noted, the statement in our previous response referring to “the provision of social support” was inaccurate and resulted from an oversight during manuscript revision. The outcome of the present study is incident oral frailty, not the provision of social support. We sincerely apologize for this error and have corrected the description accordingly.*

*We also agree with the reviewer that the JAGES dataset includes community-level social capital indicators and possesses a hierarchical structure that is suitable for multilevel modeling, as demonstrated in previous JAGES studies, including those by Saito et al. Therefore, the absence of multilevel analyses in the present study should not be interpreted as a limitation arising from data availability or data structure.*

*Our decision to employ conventional logistic regression models was a methodological choice. Following the conceptual revision of the manuscript in response to Comment 1, the primary objective of the study was to examine whether the association between socioeconomic status and incident oral frailty differed according to levels of social capital, with particular emphasis on assessing effect modification through interaction analyses. To address this objective and to facilitate interpretation and comparison with previous studies using similar analytical approaches, we employed conventional logistic regression models including interaction terms rather than multilevel models. We acknowledge that multilevel models could also be used to examine cross-level interactions; however, our intention in the present study was to provide an initial epidemiological assessment of effect modification using a parsimonious and readily interpretable modeling framework. Nevertheless, we agree that multilevel modeling could provide a more rigorous assessment of contextual effects by explicitly accounting for clustering within municipalities and simultaneously estimating individual- and community-level contributions.*

*Based on the reviewer’s comment, we have revised the limitation section (Lines 511-515) to clarify that the use of conventional regression models reflected an analytical choice rather than a constraint imposed by the data structure.*

**Comment 3：WEIGHTING FOR COMMUNITY-LEVEL INDICATORS**

The authors clarify that the weights are derived from factor loadings in the original JAGES scale development. I thank them for pointing to the methodological documentation. However, this raises a methodological question that the revised manuscript does not address: if the weights are factor loadings, the principled approach is to compute factor scores (e.g., regression-based or Bartlett scores from a measurement model fitted to the data), which is the approach taken in Saito et al. original work. A weighted composite using loadings as coefficients is an approximation that does not account for residual variance or the uncertainty in the factor structure. The authors should either justify why a weighted composite is preferred over factor scores in this specific context or acknowledge this as an additional methodological limitation if they are not able to estimate factor scores from a measurement model and re-run the analyses. At present, the community-level social capital measurement departs from the referenced scale development without justification.

***Response 3:***

*Thank you very much for this insightful methodological comment. We appreciate the reviewer’s careful consideration of the measurement approach used for the community-level social capital indicators.*

*We agree that, from a psychometric perspective, factor scores derived from a fitted measurement model (e.g., regression-based or Bartlett factor scores) represent a more rigorous approach because they explicitly account for factor structure and residual variance. We also acknowledge that this approach was adopted in the original scale development work by Saito et al.*

*In the present study, however, our objective was not to develop or re-estimate the social capital measurement model but rather to apply previously established community-level social capital indicators within an epidemiological investigation of oral frailty. Therefore, following prior JAGES studies, we constructed weighted composite scores using the published factor loadings reported by Saito et al. This approach enabled us to maintain consistency with the established operationalization of community-level social capital and to facilitate comparability with previous studies using the same indicators.*

*Nevertheless, we agree that weighted composite scores based on published factor loadings are an approximation of latent factor scores and do not fully account for residual variance, measurement error, or uncertainty in the underlying factor structure. As a result, the community-level social capital indicators used in this study may have been measured less precisely than factor scores estimated directly from a fitted measurement model. Accordingly, we have added this issue to the Limitations section and noted that the observed associations should be interpreted with appropriate caution.　In response to the reviewer’s suggestion, we have added the following statement to the Limitations section (Lines 515–519).*

Beyond the major methodological concerns above, I raise attention about some smaller issues for the next revision:

**Comment 4-1**：

Several of the authors' clarifications, like the national equivalent disposable household income figures underlying the 2-million-yen threshold (2.653M in 2018; 2.794M in 2020), and the rationale for the post-hoc power analysis are informative but are not reflected in the manuscript. The income reference values and source should be added to Lines 250–253 so that international readers need not consult the response letter.

***Response 4-1：***

*Thank you for this suggestion. We agree and have added the national equivalent disposable household income values and their source to Lines 236–243:*

(Line 236-243)” These categories were defined with reference to the average equivalent disposable household income among households with individuals aged 65 years or older in Japan, as well as the distribution of equivalent household income in the study sample. According to the National Survey of Family Income and Expenditure, the average equivalent disposable household income for households with individuals aged 65 or older was 2.653 million yen in 2018 and 2.794 million yen in 2020.”

**Comment 4-2：**

The authors' statement that excluded participants were older and in poorer health is helpful, but this is not documented in the manuscript. Given that approximately 40,000 participants were excluded, please add either a supplementary table comparing baseline characteristics of included versus excluded participants, or a brief paragraph in the Methods or Results describing the differences, with an accompanying limitation on potential selection bias.

***Response 4-2：***

*Thank you for this valuable comment. In response, we added Supplementary Table S1 comparing the baseline characteristics of the analytic sample and excluded participants, including individuals with missing data. We also added a corresponding description to the Results section (Lines 367–372) and included an additional statement in the Limitations section (Lines 507–510) regarding the potential for selection bias.*

Results (Lines 367–372)

“Table S1 compares the baseline characteristics of the analytic sample and excluded participants, including individuals with missing data. The excluded participants were generally older and less educated than those included in the analytic sample. Specifically, the mean age of excluded participants was 75.3 years, compared with 71.7 years in the analytic sample, and the proportion with ≥13 years of education was lower among excluded participants (17.4%) than among the analytic sample (40.4%).”

Limitations (Lines 517–510)

“In addition, excluded participants were generally older and less educated than those included in the analytic sample. Therefore, selection bias resulting from participant exclusion cannot be ruled out, and the findings may be less generalisable to older adults with poorer socioeconomic profiles. ”
