# Supplementary material for: Associations between health-related social capital and oral frailty risk among older adults: A stratified analysis by socioeconomic status in the JAGES longitudinal study
Source: PLoS One. 2026 Jul 24;21(7):e0353850. doi: 10.1371/journal.pone.0353850 (PMC13399358; doi:10.1371/journal.pone.0353850)
Supplement: Peer review history

# Peer review: Decision Letter 0

**DOI:** 10.1371/journal.pone.0353850.r001

**Authors:** Ryota Sakurai

4 Feb 2026

PONE-D-25-65610

Exploring the mediating role of health-related social capital in the association between socioeconomic status and oral frailty risk among community-dwelling older adults: A longitudinal study using the JAGES

PLOS One

Dear Dr. Tadaka,

Thank you for submitting your manuscript to PLOS ONE. After careful consideration, we feel that it has merit but does not fully meet PLOS ONE’s publication criteria as it currently stands. Therefore, we invite you to submit a revised version of the manuscript that addresses the points raised during the review process.

Please submit your revised manuscript by Mar 21 2026 11:59PM. If you will need more time than this to complete your revisions, please reply to this message or contact the journal office at plosone@plos.org. When you're ready to submit your revision, log on to https://www.editorialmanager.com/pone/ and select the 'Submissions Needing Revision' folder to locate your manuscript file.

Please include the following items when submitting your revised manuscript:

- A letter that responds to each point raised by the academic editor and reviewer(s). You should upload this letter as a separate file labeled 'Response to Reviewers'.
- A marked-up copy of your manuscript that highlights changes made to the original version. You should upload this as a separate file labeled 'Revised Manuscript with Track Changes'.
- An unmarked version of your revised paper without tracked changes. You should upload this as a separate file labeled 'Manuscript'.

If you would like to make changes to your financial disclosure, please include your updated statement in your cover letter. Guidelines for resubmitting your figure files are available below the reviewer comments at the end of this letter.

If applicable, we recommend that you deposit your laboratory protocols in protocols.io to enhance the reproducibility of your results. Protocols.io assigns your protocol its own identifier (DOI) so that it can be cited independently in the future. For instructions see: https://journals.plos.org/plosone/s/submission-guidelines#loc-laboratory-protocols. Additionally, PLOS ONE offers an option for publishing peer-reviewed Lab Protocol articles, which describe protocols hosted on protocols.io. Read more information on sharing protocols at https://plos.org/protocols?utm_medium=editorial-email&utm_source=authorletters&utm_campaign=protocols.

We look forward to receiving your revised manuscript.

Kind regards,

Ryota Sakurai, Ph.D.

Academic Editor

PLOS One

Journal Requirements:

When submitting your revision, we need you to address these additional requirements.

1. Please ensure that your manuscript meets PLOS ONE's style requirements, including those for file naming. The PLOS ONE style templates can be found at

https://journals.plos.org/plosone/s/file?id=wjVg/PLOSOne_formatting_sample_main_body.pdf and

https://journals.plos.org/plosone/s/file?id=ba62/PLOSOne_formatting_sample_title_authors_affiliations.pdf

2. Thank you for stating the following financial disclosure:

“This study used data from JAGES (the Japan Gerontological Evaluation Study). This study was supported by Grant-in-Aid for Scientific Research (19K02200, 20H00557, 20H03954, 20K02176,20K10540, 20K13721, 20K19534, 21H00792, 21H03196, 21K02001, 21K10323, 21K11108, 21K17302, 21K17308, 21K17322, 22H00934, 22H03299, 22J00662, 22J01409, 22K01434, 22K04450, 22K10564, 22K11101, 22K13558, 22K17265, 22K17364, 22K17409, 23K16320, 23H00449, 23H03117, 23K19793, 23K21500, 23K19796, 23K2790903 from JSPS (Japan Society for the Promotion of Science), Health Labour Sciences Research Grants (19FA1012, 19FA2001, 21FA1012,22FA2001, 22FA1010, 22FG2001), the Research Funding for Longevity Sciences from National Center for Geriatrics and Gerontology (21-20), Research Institute of Science and Technology for Society (JPMJOP1831, †RISTEX, JPMJRX21K6) from the Japan Science and Technology (JST), a grant from Japan Health Promotion & Fitness Foundation, contribution by Department of Active Ageing, Niigata University Graduate School of Medical and Dental Sciences (donated by Tokamachi city, Niigata), TMDU priority research areas grant and National Research Institute for Earth Science and Disaster Resilience.”

Please state what role the funders took in the study.  If the funders had no role, please state: "The funders had no role in study design, data collection and analysis, decision to publish, or preparation of the manuscript."

If this statement is not correct you must amend it as needed.

Please include this amended Role of Funder statement in your cover letter; we will change the online submission form on your behalf.

3. We note that you have indicated that there are restrictions to data sharing for this study. PLOS only allows data to be available upon request if there are legal or ethical restrictions on sharing data publicly. For more information on unacceptable data access restrictions, please see http://journals.plos.org/plosone/s/data-availability#loc-unacceptable-data-access-restrictions.

Before we proceed with your manuscript, please address the following prompts:

a) If there are ethical or legal restrictions on sharing a de-identified data set, please explain them in detail (e.g., data contain potentially identifying or sensitive patient information, data are owned by a third-party organization, etc.) and who has imposed them (e.g., a Research Ethics Committee or Institutional Review Board, etc.). Please also provide contact information for a data access committee, ethics committee, or other institutional body to which data requests may be sent.

b) If there are no restrictions, please upload the minimal anonymized data set necessary to replicate your study findings to a stable, public repository and provide us with the relevant URLs, DOIs, or accession numbers. For a list of recommended repositories, please see

https://journals.plos.org/plosone/s/recommended-repositories. You also have the option of uploading the data as Supporting Information files, but we would recommend depositing data directly to a data repository if possible.

We will update your Data Availability statement on your behalf to reflect the information you provide.

4. If the reviewer comments include a recommendation to cite specific previously published works, please review and evaluate these publications to determine whether they are relevant and should be cited. There is no requirement to cite these works unless the editor has indicated otherwise.

Additional Editor Comments :

Two reviewers have raised several critical concerns, and I ask the authors to respond to all of these points. In addition to the reviewers’ comments, I would like to raise the following issues:

1) It is unclear why the authors concluded that individual‑level social capital mitigates SES‑related disparities in oral frailty risk. Which specific results support this conclusion? In the Introduction, the manuscript refers to “mediation,” and the title also implies a mediating effect. However, the study does not appear to assess mediation in a systematic manner, such as through mediation analysis. Please clarify how the authors evaluated a mediating effect and which findings indicate that social capital mediates, rather than moderates, the association between SES and oral frailty risk.

2) The meaning of the reported sensitivity (0.94) and specificity (0.67) on page 9, line 205, is unclear. Sensitivity and specificity of what measure or classification? Additionally, it is not clear why the oral frailty risk score was calculated using a logistic regression equation rather than using the raw score. Please explain the rationale for this approach.

3) There are instances of overstatement and insufficient description. For example, line 412 states that “In the high‑SES group, community‑level social capital had no significant effect on oral frailty.” However, this appears to apply only to the civic participation score. In the low‑SES group, the social cohesion score also shows no significant effect on oral frailty. In the original manuscript, since the results derived from multiple measurement items are being interpreted conveniently, please clarify the interpretation of the results.

[Note: HTML markup is below. Please do not edit.]

Reviewers' comments:

Reviewer's Responses to Questions

**Comments to the Author**

1. Is the manuscript technically sound, and do the data support the conclusions?

The manuscript must describe a technically sound piece of scientific research with data that supports the conclusions. Experiments must have been conducted rigorously, with appropriate controls, replication, and sample sizes. The conclusions must be drawn appropriately based on the data presented.

Reviewer #1: Yes

Reviewer #2: Partly

**********

2. Has the statistical analysis been performed appropriately and rigorously?

Reviewer #1: Yes

Reviewer #2: No

**********

3. Have the authors made all data underlying the findings in their manuscript fully available?

The PLOS Data policy requires authors to make all data underlying the findings described in their manuscript fully available without restriction, with rare exception (please refer to the Data Availability Statement in the manuscript PDF file). The data should be provided as part of the manuscript or its supporting information, or deposited to a public repository. For example, in addition to summary statistics, the data points behind means, medians and variance measures should be available. If there are restrictions on publicly sharing data—e.g. participant privacy or use of data from a third party—those must be specified.

Reviewer #1: Yes

Reviewer #2: No

**********

4. Is the manuscript presented in an intelligible fashion and written in standard English?

PLOS ONE does not copyedit accepted manuscripts, so the language in submitted articles must be clear, correct, and unambiguous. Any typographical or grammatical errors should be corrected at revision, so please note any specific errors here.

Reviewer #1: Yes

Reviewer #2: Yes

**********

5. Review Comments to the Author

Please use the space provided to explain your answers to the questions above. You may also include additional comments for the author, including concerns about dual publication, research ethics, or publication ethics. (Please upload your review as an attachment if it exceeds 20,000 characters)

Reviewer #1: Thank you for the opportunity to review this article.

This longitudinal cohort study examined the mediating role of health-related social capital in the relationship between socioeconomic status (SES) and oral frailty risk among community-dwelling older adults in Japan, using data from the Japan Gerontological Evaluation Study (JAGES). The study is well-structured and based on a large, nationally representative dataset, and the topic is timely. Below are suggestions to improve clarity, transparency, and scientific validity, particularly with respect to observational study reporting guidelines.

First, the paper mostly aligns with STROBE reporting standards, but a few checklist items could be improved for completeness and transparency. The abstract includes a clear summary of the design and findings, though the specific mention of the longitudinal nature of the cohort could be made more explicit. A flow diagram is appropriately provided (Figure on page 35), and descriptive statistics and model outputs are clearly presented (Tables 1–7, pages 14–18). However, information on missing data for each variable (STROBE Item 14b) is not fully reported. It would be helpful to specify the extent of missingness for key variables and how they were handled, particularly given the large initial sample and substantial exclusions during follow-up.

Second, the use of a validated predictive model to estimate oral frailty risk at follow-up is appropriate, but the model itself is based on non-clinical indicators (e.g., swallowing function, chewing difficulty). The distinction between predicted risk and actual clinical diagnosis should be more clearly emphasized, and the outcome measure described consistently throughout the manuscript. This is important for construct validity.

Third, while the SES measure (equivalent income) and social capital dimensions are well defined, the rationale for dichotomizing SES at 2 million yen could be clarified. Additionally, although the authors note adjustment for potential confounders, the selection criteria for covariates and whether any sensitivity analyses were conducted to test alternative specifications (STROBE Item 12e, 17) are not stated. Reporting on robustness checks would strengthen the internal validity of the findings.

Fourth, the stratified analysis by SES adds value, but subgroup sizes and event counts in each group should be reported directly to allow readers to assess precision. Furthermore, interpretation of effect sizes should be presented cautiously, especially given the relatively small differences in mean scores and odds ratios near 1.0 (e.g., OR 0.947, 95% CI: 0.932–0.963 for individual-level social capital).

Fifth, the discussion is generally well reasoned, and limitations are acknowledged. However, some conclusions remain slightly overextended in terms of policy recommendations. For example, while the study supports associations between social capital and oral frailty risk, statements that interventions “may contribute to the sustainability of social security systems” or that social capital “reduces risk” could be rephrased more conservatively, given the observational nature of the data.

Sixth, the manuscript does not report whether any efforts were made to address potential bias, including unmeasured confounding or selection bias due to loss to follow-up (STROBE Item 9, 12d). While the exclusion criteria are thorough, the potential for differential loss should be discussed in more detail.

Seventh, the statistical methods are clearly described, and the models are adjusted for relevant demographic and health-related covariates. Odds ratios and confidence intervals are presented appropriately. However, as noted above, the paper would benefit from sensitivity analyses—such as alternative SES thresholds, interaction terms, or models excluding certain predictors—to assess robustness.

Overall, this is a rigorous and policy-relevant study that offers valuable insight into the intersection of SES, social capital, and aging-related oral health. A few clarifications on methodological decisions and greater transparency in reporting would enhance the manuscript further. I hope the authors find these suggestions helpful.

Reviewer #2: I thank the authors for submitting this manuscript addressing an important and timely topic. The investigation of social capital as a potential buffer against oral frailty risk among older adults with lower socioeconomic status represents a meaningful contribution to the gerontological and public health literature. The use of longitudinal data from a large, well-established cohort study is commendable, and the research question has clear implications for healthy aging policies.

While the manuscript addresses a relevant question with appropriate data, several conceptual and methodological issues require attention before the work can be considered for publication. Most critically, there is a fundamental inconsistency between the stated aim (examining "mediating effects") and the analytical approach employed (stratification by SES). Stratification examines whether the association between social capital and oral frailty differs across SES strata and this is effect modification or moderation, not mediation. Throughout the manuscript, there is no mention of formal mediation analysis techniques (e.g., causal mediation analysis, structural equation modeling). I strongly recommend that the authors either (a) revise the title, aims, and framing to accurately reflect a moderation analysis, or (b) conduct and report proper mediation analyses if the original research question is indeed about mediation pathways.

Additionally, the authors describe their work as a "multilevel approach," which may mislead readers familiar with multilevel (hierarchical) regression models. The current analysis aggregates community-level variables and includes them as individual-level predictors in standard logistic regression, which does not account for the nested data structure (individuals within municipalities). A true multilevel model would better accommodate community-level effects and the clustering of participants. If such modeling was not feasible, this should be acknowledged as a limitation, with recommendations for future research.

Bellow, I present section-specific questions and some comments aimed at helping improve the reporting of the investigation.

ABSTRACT: The phrasing "longitudinal data from the Japan Gerontological Evaluation Study (JAGES) collected in 2019 and 2022" is imprecise. "In 2019 and 2022" denotes two discrete time points, not a range. Consider revising to "collected at baseline (2019) and follow-up (2022)" or similar phrasing that accurately reflects the two-wave design.

INTRODUCTION

--- The manuscript would benefit from a clear definition of "healthy life expectancy," briefly characterizing what is understood as health during this life stage, particularly in the Japanese societal context. Does it mean maintaining the integrity of physiological and psychological capacities? Is it related to individual autonomy and social integration? A brief clarification would anchor the reader's understanding.

--- I am not sure the argument that health inequalities "widen with age and become more pronounced later in life" is clearly stated. Why would that be? The mechanism or evidence supporting this claim should be briefly articulated.

--- Is the problem really "identifying effective and equitable strategies to reduce health disparities regardless of SES," or is it more precisely to account for the influences of SES on said health disparities? The current framing seems to understate the centrality of SES as a structural determinant.

--- How does access to oral health services impact oral frailty in the context of Japan? Do older adults have universal access to dental care? Is SES relevant because only more privileged individuals are granted access to oral health care, or are there other pathways? I believe this aspect of the Japanese healthcare system should be clarified, as it may significantly blur the interpretation of SES effects.

METHODS

---Which 76 municipalities were included? Out of how many total municipalities in Japan? Providing this context would help readers assess the generalizability of findings.

--- Was a baseline sample size estimated a priori? If so, please report the parameters used.

--- How were participants randomized in municipalities with over 5,000 residents? The sampling procedure should be described in sufficient detail for replication.

--- The exclusion criteria appear very strict. While I understand the rationale for ensuring a functionally independent baseline sample, this should be acknowledged when discussing generalizability.

--- What is the exact reasoning behind the differential weighting of community-level indicators of social capital? As currently presented, the different weights seem arbitrary. In the referenced Saito et al. original scale, there is no such weighting suggestion; rather, factor scores are estimated. A clear justification should be presented for the weighting scheme used. Furthermore, in their investigation, Saito et al. use multilevel regression to model exposure and outcomes. Was this not possible in the current investigation? A multilevel model would better account for common community-level variation in social capital and the clustering of individuals within municipalities.

--- Is it possible to specify which chronic diseases were considered and how they were modeled in the regression (e.g., score range, categorization)?

--- It would be helpful if the authors could better describe attrition and missing data at baseline and follow-up, particularly for the 40,000 participants excluded from analysis. Do excluded participants differ systematically from those retained? Of those, how many presented with oral frailty risk at baseline?

--- Could the authors provide better context regarding the equivalent household income variable? Why was the 2 million yen threshold chosen for segregating SES groups? Is this based on minimum wage, an indicator of poverty, or a similar underprivileged situation in Japan? While this may be clear to those familiar with the Japanese economy, for a broader international readership it would be helpful to have additional explanation given that this is the main exposure variable.

RESULTS

--- Table 2 could be consolidated as a first or final row in Table 1, rather than presented as a separate table. Additionally, in terms of descriptive potential, an initial table might be more informative if structured by the outcome variable (oral frailty risk) rather than by SES level.

--- I would recommend reconsidering the presentation of oral frailty incidence by person-years (lines 314–315), as this metric is not explored elsewhere in the manuscript and may confuse readers given the discrete two-wave design.

--- The mean values for community-level indicators are nearly identical across SES groups (indeed, identical for reciprocity), with statistical significance likely driven by sample size rather than meaningful differences. This limited between-group variation in community-level social capital may explain the null findings for community-level moderation effects reported later, a point worth acknowledging in the discussion as a potential limitation of the analytic strategy rather than evidence that community-level social capital is unimportant.

DISCUSSION

--- I believe it would be valuable to add a sentence or two in the discussion regarding the dissipation of sex effects on oral frailty in the high-SES group across all three models. Why does being a woman appear protective among low-SES participants but not among high-SES participants? This pattern warrants interpretation.

--- In a brief PubMed search, I identified several studies with either corroborating or conflicting findings that could strengthen the discussion, particularly regarding the effects of reciprocity and civic participation on oral health outcomes in older adults. I recommend the authors consider engaging with the following works:

------ Community Dent Oral Epidemiol. 2023 Oct;51(5):879-886. doi: 10.1111/cdoe.12780.

------ PLoS One. 2015 May 18;10(5):e0125557. doi: 10.1371/journal.pone.0125557.

------ BMC Oral Health. 2022 Mar 12;22(1):68. doi: 10.1186/s12903-022-02102-8.

------ J Appl Gerontol. 2022 Mar;41(3):671-679. doi: 10.1177/07334648211028391.

------ BMC Public Health. 2025 Apr 15;25(1):1401. doi: 10.1186/s12889-025-22649-9.

**********

6. PLOS authors have the option to publish the peer review history of their article (what does this mean?). If published, this will include your full peer review and any attached files.

If you choose “no”, your identity will remain anonymous but your review may still be made public.

**Do you want your identity to be public for this peer review?**  For information about this choice, including consent withdrawal, please see our Privacy Policy.

Reviewer #1: No

Reviewer #2: **Yes:** Cézar Luquine

**********

[NOTE: If reviewer comments were submitted as an attachment file, they will be attached to this email and accessible via the submission site. Please log into your account, locate the manuscript record, and check for the action link "View Attachments". If this link does not appear, there are no attachment files.]

To ensure your figures meet our technical requirements, please review our figure guidelines: https://journals.plos.org/plosone/s/figures

You may also use PLOS’s free figure tool, NAAS, to help you prepare publication quality figures: https://journals.plos.org/plosone/s/figures#loc-tools-for-figure-preparation.

NAAS will assess whether your figures meet our technical requirements by comparing each figure against our figure specifications.

# Author comment: Author response to Decision Letter 1

**DOI:** 10.1371/journal.pone.0353850.r002

23 Mar 2026

We sincerely thank you and the reviewers for the careful evaluation of our manuscript and for providing insightful comments. We greatly appreciate the time and effort invested in the review process.

We have carefully addressed all points raised and revised the manuscript accordingly. In particular:

Mediating role of individual-level social capital: We have now conducted a formal mediation analysis using structural equation modeling (SEM) and presented the results in the revised manuscript. These results clarify which associations support the conclusion that individual-level social capital mediates SES-related disparities in oral frailty risk, clearly distinguishing mediation from moderation.

Sensitivity and specificity: We have clarified that the reported sensitivity (0.94) and specificity (0.67) refer to the logistic regression-based classification of oral frailty risk. We also provide a rationale for calculating the oral frailty risk score using the logistic regression equation rather than the raw score, highlighting its predictive accuracy and interpretability.

Overstatement and interpretation of results: We have revised the manuscript to avoid overgeneralization. The description of high-SES and low-SES group results now specifies the associations for each component of social capital (e.g., civic participation and social cohesion), providing a clearer and more accurate interpretation of the findings.

Role of funders: The funders had no role in study design, data collection and analysis, decision to publish, or preparation of the manuscript.

All data are from the Japan Agency for Gerontological Evaluation Study (URL: https://www.jages.net/). Data cannot be shared publicly because all JAGES datasets have ethical or legal restrictions for public deposition due to inclusion of sensitive information from the human participants. All inquiries on the data are to be addressed at JAGES data management committee via e-mail: dataadmin.ml@jages.net.

We believe that these revisions, including the SEM-based formal mediation analysis, substantially improve the clarity, rigor, and transparency of our study. We hope that the revised manuscript now meets PLOS ONE’s publication criteria and would like to thank you again for your constructive feedback.

Attachment Submitted filename: 260319Response to Reviewers.docx

# Peer review: Decision Letter 1

**DOI:** 10.1371/journal.pone.0353850.r003

**Authors:** Ryota Sakurai

28 Apr 2026

PONE-D-25-65610R1

Exploring the mediating role of health-related social capital in the association between socioeconomic status and oral frailty risk among community-dwelling older adults: A longitudinal study using the JAGES

PLOS One

Dear Dr. Tadaka,

Thank you for submitting your manuscript to PLOS ONE. After careful consideration, we feel that it has merit but does not fully meet PLOS ONE’s publication criteria as it currently stands. Therefore, we invite you to submit a revised version of the manuscript that addresses the points raised during the review process.

Please submit your revised manuscript by Jun 12 2026 11:59PM. If you will need more time than this to complete your revisions, please reply to this message or contact the journal office at plosone@plos.org. When you're ready to submit your revision, log on to https://www.editorialmanager.com/pone/ and select the 'Submissions Needing Revision' folder to locate your manuscript file.

Please include the following items when submitting your revised manuscript:

- A letter that responds to each point raised by the academic editor and reviewer(s). You should upload this letter as a separate file labeled 'Response to Reviewers'.
- A marked-up copy of your manuscript that highlights changes made to the original version. You should upload this as a separate file labeled 'Revised Manuscript with Track Changes'.
- An unmarked version of your revised paper without tracked changes. You should upload this as a separate file labeled 'Manuscript'.

If you would like to make changes to your financial disclosure, please include your updated statement in your cover letter. Guidelines for resubmitting your figure files are available below the reviewer comments at the end of this letter.

If applicable, we recommend that you deposit your laboratory protocols in protocols.io to enhance the reproducibility of your results. Protocols.io assigns your protocol its own identifier (DOI) so that it can be cited independently in the future. For instructions see: https://journals.plos.org/plosone/s/submission-guidelines#loc-laboratory-protocols. Additionally, PLOS ONE offers an option for publishing peer-reviewed Lab Protocol articles, which describe protocols hosted on protocols.io. Read more information on sharing protocols at https://plos.org/protocols?utm_medium=editorial-email&utm_source=authorletters&utm_campaign=protocols.

As the corresponding author, your ORCID iD is verified in the submission system and will appear in the published article. PLOS supports the use of ORCID, and we encourage all coauthors to register for an ORCID iD and use it as well. Please encourage your coauthors to verify their ORCID iD within the submission system before final acceptance, as unverified ORCID iDs will not appear in the published article. *Only*  the individual author can complete the verification step; PLOS staff *cannot*  verify ORCID iDs on behalf of authors.

We look forward to receiving your revised manuscript.

Kind regards,

Ryota Sakurai, Ph.D.

Academic Editor

PLOS One

Journal Requirements:

If the reviewer comments include a recommendation to cite specific previously published works, please review and evaluate these publications to determine whether they are relevant and should be cited. There is no requirement to cite these works unless the editor has indicated otherwise.

Additional Editor Comments:

Thank you for your efforts to improve the manuscript. However, the reviewer still has several concerns that must be addressed. Please carefully review the comments and provide a point-by-point response to all of them.

[Note: HTML markup is below. Please do not edit.]

Reviewers' comments:

Reviewer's Responses to Questions

**Comments to the Author**

1. If the authors have adequately addressed your comments raised in a previous round of review and you feel that this manuscript is now acceptable for publication, you may indicate that here to bypass the “Comments to the Author” section, enter your conflict of interest statement in the “Confidential to Editor” section, and submit your "Accept" recommendation.

Reviewer #2: (No Response)

**********

2. Is the manuscript technically sound, and do the data support the conclusions?

The manuscript must describe a technically sound piece of scientific research with data that supports the conclusions. Experiments must have been conducted rigorously, with appropriate controls, replication, and sample sizes. The conclusions must be drawn appropriately based on the data presented.

Reviewer #2: Partly

**********

3. Has the statistical analysis been performed appropriately and rigorously?

Reviewer #2: No

**********

4. Have the authors made all data underlying the findings in their manuscript fully available?

The PLOS Data policy requires authors to make all data underlying the findings described in their manuscript fully available without restriction, with rare exception (please refer to the Data Availability Statement in the manuscript PDF file). The data should be provided as part of the manuscript or its supporting information, or deposited to a public repository. For example, in addition to summary statistics, the data points behind means, medians and variance measures should be available. If there are restrictions on publicly sharing data—e.g. participant privacy or use of data from a third party—those must be specified.

Reviewer #2: No

**********

5. Is the manuscript presented in an intelligible fashion and written in standard English?

PLOS ONE does not copyedit accepted manuscripts, so the language in submitted articles must be clear, correct, and unambiguous. Any typographical or grammatical errors should be corrected at revision, so please note any specific errors here.

Reviewer #2: Yes

**********

6. Review Comments to the Author

Please use the space provided to explain your answers to the questions above. You may also include additional comments for the author, including concerns about dual publication, research ethics, or publication ethics. (Please upload your review as an attachment if it exceeds 20,000 characters)

Reviewer #2: I thank the authors for their thorough point-by-point response and for the substantive revisions made to the manuscript. Several of my initial concerns have been appropriately addressed, including the clarification of healthy life expectancy, the addition of the cumulative advantage/disadvantage framework, the explanation of the Japanese universal healthcare context, and the rationale for the 2-million-yen SES threshold. These revisions improve the manuscript.

However, three of my central methodological concerns have not been adequately resolved, and a few of the smaller points would benefit from revisions being made visible in the manuscript itself rather than only in the response letter. I detail these below.

MEDIATION ANALYSIS IS INCONSISTENT WITHIT THE MANUSCRIPT:

Based on Figure 2, for instance, the path diagram depicts SES as the exposure, with direct paths from SES to education, household composition, sex, and oral frailty risk, and an indirect path from SES through community-level civic participation to oral frailty risk. This is, structurally, a mediation model in which SES is the exposure. However, the figure title ("Mediating structure of Community-level Civic participation in Low-SES for Oral frailty risk") and the methods text ("SEM was conducted for both the low-SES and high-SES groups to analyze the mediating structure") describe stratified estimation within SES subgroups. These are incompatible: SES cannot simultaneously be the exposure in a fitted path model and the variable used to split the sample into two separate models. If the mediation model in Figure 2 was estimated within the low-SES subsample, the SES → civic participation path cannot be meaningfully estimated because within-subsample variance in SES is restricted by the stratification itself. Conversely, if SES is a functioning exposure variable in the model, then the "in Low-SES" labeling is inaccurate. The authors should clarify exactly what was estimated.

Beyond this internal inconsistency, the model specification in Figure 2 raises concerns even taken on its own terms. The paths SES → sex and SES → education cannot be interpreted causally, since sex is determined at birth and educational attainment is typically completed decades before SES measurement in this cohort. This suggests the arrows are being used as statistical regression paths rather than directed causal relationships, which is required by mediation analysis for the indirect effect to have meaning. Additionally, modeling covariates (education, household composition, sex) as outcomes of the exposure rather than as exogenous adjustment variables changes the meaning of the direct and indirect effects in ways the manuscript does not address. Covariates should typically be modeled as exogenous predictors of the mediator and outcome (Valeri & VanderWeele, 2013, Psychological Methods, 18(2), 137–150).

Finally, no decomposition of total, direct, and indirect effects is reported, which is the purpose of a mediation analysis. The authors' statement that the SEM findings "are consistent with the associations observed in the logistic regression analyses" suggests the SEM is being used descriptively to recover the regression estimate rather than to quantify mediation pathways.

Two further technical issues warrant clarification. First, oral frailty risk is a binary outcome, but the estimator used in Amos is not specified; default maximum likelihood estimation with a binary outcome produces somewhat biased estimates. Second, the decision to fit SEM only for social capital variables significant in the low-SES logistic regression is a post-hoc selection step that compromises interpretation of the corresponding high-SES models, which are estimated conditionally on results from the other stratum.

I recommend the authors take one of two paths. Either (a) fit a unified mediation model with SES as exposure on the full sample and report indirect, direct, and total effect decomposition, or (b) remove the mediation framing entirely, revise the title, aims, and discussion to present this as a moderation/effect modification study, and report the stratified analyses transparently as such. The current version attempts to frame a stratified analysis as mediation, which will mislead readers.

MULTILEVEL MODELLING:

The removal of "multilevel approach" from the manuscript is appropriate, and the added limitation at Lines 554–558 is a reasonable compromise. However, the rationale provided in the response letter is inconsistent with the study design. The authors state that "our primary analytical focus was to examine the association between individual characteristics and the provision of social support." Social support provision is not, as I understand it, the outcome of this study; oral frailty risk is. This phrasing suggests either a drafting error or that this justification was adapted from another context, and I would ask the authors to clarify.

More substantively, the authors note that Saito et al. employed multilevel regression with the JAGES community-level social capital indicators, which implies that the data structure does, in fact, support multilevel modeling. The constraint is therefore analytical choice rather than data availability. Thys, justification as currently written is not coherent and should be revised.

WEIGHTING FOR COMMUNITY-LEVEL INDICATORS

The authors clarify that the weights are derived from factor loadings in the original JAGES scale development. I thank them for pointing to the methodological documentation. However, this raises a methodological question that the revised manuscript does not address: if the weights are factor loadings, the principled approach is to compute factor scores (e.g., regression-based or Bartlett scores from a measurement model fitted to the data), which is the approach taken in Saito et al. original work. A weighted composite using loadings as coefficients is an approximation that does not account for residual variance or the uncertainty in the factor structure. The authors should either justify why a weighted composite is preferred over factor scores in this specific context or acknowledge this as an additional methodological limitation if they are not able to estimate factor scores from a measurement model and re-run the analyses. At present, the community-level social capital measurement departs from the referenced scale development without justification.

Beyond the major methodological concerns above, I raise attention about some smaller issues for the next revision:

1. Several of the authors' clarifications, like the national equivalent disposable household income figures underlying the 2-million-yen threshold (2.653M in 2018; 2.794M in 2020), and the rationale for the post-hoc power analysis are informative but are not reflected in the manuscript. The income reference values and source should be added to Lines 250–253 so that international readers need not consult the response letter.

2. The authors' statement that excluded participants were older and in poorer health is helpful, but this is not documented in the manuscript. Given that approximately 40,000 participants were excluded, please add either a supplementary table comparing baseline characteristics of included versus excluded participants, or a brief paragraph in the Methods or Results describing the differences, with an accompanying limitation on potential selection bias.

The concerns raised above remain the core issues preventing publication. Addressing them substantively, rather than through reframing or additional caveats, is essential before the manuscript can be considered further. I hope these comments are useful to the authors in strengthening what is an important and timely investigation.

**********

7. PLOS authors have the option to publish the peer review history of their article (what does this mean?). If published, this will include your full peer review and any attached files.

If you choose “no”, your identity will remain anonymous but your review may still be made public.

**Do you want your identity to be public for this peer review?**  For information about this choice, including consent withdrawal, please see our Privacy Policy.

Reviewer #2: **Yes:** Cézar Luquine

**********

[NOTE: If reviewer comments were submitted as an attachment file, they will be attached to this email and accessible via the submission site. Please log into your account, locate the manuscript record, and check for the action link "View Attachments". If this link does not appear, there are no attachment files.]

To ensure your figures meet our technical requirements, please review our figure guidelines: https://journals.plos.org/plosone/s/figures

You may also use PLOS’s free figure tool, NAAS, to help you prepare publication quality figures: https://journals.plos.org/plosone/s/figures#loc-tools-for-figure-preparation.

NAAS will assess whether your figures meet our technical requirements by comparing each figure against our figure specifications.

# Author comment: Author response to Decision Letter 2

**DOI:** 10.1371/journal.pone.0353850.r004

11 Jun 2026

To Reviewer 2

Comment 1　MEDIATION ANALYSIS IS INCONSISTENT WITH THE MANUSCRIPT:

Based on Figure 2, for instance, the path diagram depicts SES as the exposure, with direct paths from SES to education, household composition, sex, and oral frailty risk, and an indirect path from SES through community-level civic participation to oral frailty risk. This is, structurally, a mediation model in which SES is the exposure. However, the figure title ("Mediating structure of Community-level Civic participation in Low-SES for Oral frailty risk") and the methods text ("SEM was conducted for both the low-SES and high-SES groups to analyze the mediating structure") describe stratified estimation within SES subgroups. These are incompatible: SES cannot simultaneously be the exposure in a fitted path model and the variable used to split the sample into two separate models. If the mediation model in Figure 2 was estimated within the low-SES subsample, the SES → civic participation path cannot be meaningfully estimated because within-subsample variance in SES is restricted by the stratification itself. Conversely, if SES is a functioning exposure variable in the model, then the "in Low-SES" labeling is inaccurate. The authors should clarify exactly what was estimated.

Beyond this internal inconsistency, the model specification in Figure 2 raises concerns even when taken on its own terms. The paths SES → sex and SES → education cannot be interpreted causally, since sex is determined at birth and educational attainment is typically completed decades before SES measurement in this cohort. This suggests the arrows are being used as statistical regression paths rather than directed causal relationships, which is required by mediation analysis for the indirect effect to have meaning. Additionally, modeling covariates (education, household composition, sex) as outcomes of the exposure rather than as exogenous adjustment variables changes the meaning of the direct and indirect effects in ways the manuscript does not address. Covariates should typically be modeled as exogenous predictors of the mediator and outcome (Valeri & VanderWeele, 2013, Psychological Methods, 18(2), 137–150).

Finally, no decomposition of total, direct, and indirect effects is reported, which is the purpose of a mediation analysis. The authors' statement that the SEM findings "are consistent with the associations observed in the logistic regression analyses" suggests the SEM is being used descriptively to recover the regression estimate rather than to quantify mediation pathways.

Two further technical issues warrant clarification. First, oral frailty risk is a binary outcome, but the estimator used in Amos is not specified; default maximum likelihood estimation with a binary outcome produces somewhat biased estimates. Second, the decision to fit SEM only for social capital variables significant in the low-SES logistic regression is a post-hoc selection step that compromises interpretation of the corresponding high-SES models, which are estimated conditionally on results from the other stratum.　I recommend the authors take one of two paths. Either

(a) fit a unified mediation model with SES as exposure on the full sample and report indirect, direct, and total effect decomposition, or

(b) remove the mediation framing entirely, revise the title, aims, and discussion to present this as a moderation/effect modification study, and report the stratified analyses transparently as such.

Response 1�

We sincerely thank the reviewer for the thorough and insightful evaluation of our manuscript and for the constructive comments regarding the inconsistency between the mediation framework and the analyses presented. We are also grateful for the additional clarification provided in the subsequent review. After carefully revisiting the reviewer’s comments, we recognized that our previous revision did not fully address the conceptual concerns regarding the mediation framework. Upon reconsideration, we fully agree that there were conceptual and methodological inconsistencies in framing the study as a mediation analysis. In particular, we agree that SES cannot simultaneously function as an exposure variable within a mediation model and as the variable used to stratify the study population. We also acknowledge that the SEM presented in the previous version did not provide sufficient evidence to support a mediation interpretation.

Accordingly, following the reviewer’s recommendation (b), we substantially restructured the manuscript. Specifically, we completely removed the mediation framework and revised the Title, Abstract, Introduction, Study Aims, Methods, Results, and Discussion sections (lines 1–3, 25–29, 36-39, 46-51, 54-57, 118-123, 130-136, 148–173, 236–250, 327–334, 391-394, 404-407, 415-418, 427-430, 453-459, 446–491) to present the study as an investigation of moderation/effect modification. Consistent with this conceptual revision, all SEM analyses, figures, and related descriptions have been removed from the manuscript. Furthermore, in accordance with the reviewer’s recommendation to “report the stratified analyses transparently as such,” we have explicitly reclassified the previously conducted analyses in the low-SES and high-SES groups as stratified analyses rather than mediation analyses. In addition, we have reframed the study to examine whether the association between SES and incident oral frailty differs according to levels of social capital.

To formally assess effect modification, we additionally conducted logistic regression analyses using the full study population. Specifically, we fitted models including SES, social capital (SC), an SES × SC interaction term, and relevant covariates (Frailty = SES + SC + SES × SC + covariates). The results showed that the interaction between SES and individual-level civic participation was statistically significant, indicating that the association between SES and incident oral frailty differed according to the level of individual-level civic participation. These findings support the effect-modification hypothesis and are consistent with the results of the stratified analyses. Importantly, this revised analytical framework avoids the conceptual inconsistencies associated with the previous mediation framework and more appropriately addresses the reviewer’s concern regarding the interpretation of the relationship between SES, social capital, and oral frailty.

The results of these analyses have been added to Tables 3–6, and lines 391-394, 404-407, 415-418, 427-430, and 453-459 have been revised accordingly.

Comment 2　MULTILEVEL MODELLING:

The removal of "multilevel approach" from the manuscript is appropriate, and the added limitation at Lines 554–558 is a reasonable compromise. However, the rationale provided in the response letter is inconsistent with the study design. The authors state that "our primary analytical focus was to examine the association between individual characteristics and the provision of social support." Social support provision is not, as I understand it, the outcome of this study; oral frailty risk is. This phrasing suggests either a drafting error or that this justification was adapted from another context, and I would ask the authors to clarify.

More substantively, the authors note that Saito et al. employed multilevel regression with the JAGES community-level social capital indicators, which implies that the data structure does, in fact, support multilevel modeling. The constraint is therefore analytical choice rather than data availability. Thys, justification as currently written is not coherent and should be revised.

Response 2�

We appreciate the reviewer’s careful reading of both the manuscript and our response letter. As the reviewer correctly noted, the statement in our previous response referring to “the provision of social support” was inaccurate and resulted from an oversight during manuscript revision. The outcome of the present study is incident oral frailty, not the provision of social support. We sincerely apologize for this error and have corrected the description accordingly.

We also agree with the reviewer that the JAGES dataset includes community-level social capital indicators and possesses a hierarchical structure that is suitable for multilevel modeling, as demonstrated in previous JAGES studies, including those by Saito et al. Therefore, the absence of multilevel analyses in the present study should not be interpreted as a limitation arising from data availability or data structure.

Our decision to employ conventional logistic regression models was a methodological choice. Following the conceptual revision of the manuscript in response to Comment 1, the primary objective of the study was to examine whether the association between socioeconomic status and incident oral frailty differed according to levels of social capital, with particular emphasis on assessing effect modification through interaction analyses. To address this objective and to facilitate interpretation and comparison with previous studies using similar analytical approaches, we employed conventional logistic regression models including interaction terms rather than multilevel models. We acknowledge that multilevel models could also be used to examine cross-level interactions; however, our intention in the present study was to provide an initial epidemiological assessment of effect modification using a parsimonious and readily interpretable modeling framework. Nevertheless, we agree that multilevel modeling could provide a more rigorous assessment of contextual effects by explicitly accounting for clustering within municipalities and simultaneously estimating individual- and community-level contributions.

Based on the reviewer’s comment, we have revised the limitation section (Lines 511-515) to clarify that the use of conventional regression models reflected an analytical choice rather than a constraint imposed by the data structure.

Comment 3�WEIGHTING FOR COMMUNITY-LEVEL INDICATORS

The authors clarify that the weights are derived from factor loadings in the original JAGES scale development. I thank them for pointing to the methodological documentation. However, this raises a methodological question that the revised manuscript does not address: if the weights are factor loadings, the principled approach is to compute factor scores (e.g., regression-based or Bartlett scores from a measurement model fitted to the data), which is the approach taken in Saito et al. original work. A weighted composite using loadings as coefficients is an approximation that does not account for residual variance or the uncertainty in the factor structure. The authors should either justify why a weighted composite is preferred over factor scores in this specific context or acknowledge this as an additional methodological limitation if they are not able to estimate factor scores from a measurement model and re-run the analyses. At present, the community-level social capital measurement departs from the referenced scale development without justification.

Response 3:

Thank you very much for this insightful methodological comment. We appreciate the reviewer’s careful consideration of the measurement approach used for the community-level social capital indicators.

We agree that, from a psychometric perspective, factor scores derived from a fitted measurement model (e.g., regression-based or Bartlett factor scores) represent a more rigorous approach because they explicitly account for factor structure and residual variance. We also acknowledge that this approach was adopted in the original scale development work by Saito et al.

In the present study, however, our objective was not to develop or re-estimate the social capital measurement model but rather to apply previously established community-level social capital indicators within an epidemiological investigation of oral frailty. Therefore, following prior JAGES studies, we constructed weighted composite scores using the published factor loadings reported by Saito et al. This approach enabled us to maintain consistency with the established operationalization of community-level social capital and to facilitate comparability with previous studies using the same indicators.

Nevertheless, we agree that weighted composite scores based on published factor loadings are an approximation of latent factor scores and do not fully account for residual variance, measurement error, or uncertainty in the underlying factor structure. As a result, the community-level social capital indicators used in this study may have been measured less precisely than factor scores estimated directly from a fitted measurement model. Accordingly, we have added this issue to the Limitations section and noted that the observed associations should be interpreted with appropriate caution.　In response to the reviewer’s suggestion, we have added the following statement to the Limitations section (Lines 515–519).

Beyond the major methodological concerns above, I raise attention about some smaller issues for the next revision:

Comment 4-1�

Several of the authors' clarifications, like the national equivalent disposable household income figures underlying the 2-million-yen threshold (2.653M in 2018; 2.794M in 2020), and the rationale for the post-hoc power analysis are informative but are not reflected in the manuscript. The income reference values and source should be added to Lines 250–253 so that international readers need not consult the response letter.

Response 4-1�

Thank you for this suggestion. We agree and have added the national equivalent disposable household income values and their source to Lines 236–243:

(Line 236-243)” These categories were defined with reference to the average equivalent disposable household income among households with individuals aged 65 years or older in Japan, as well as the distribution of equivalent household income in the study sample. According to the National Survey of Family Income and Expenditure, the average equivalent disposable household income for households with individuals aged 65 or older was 2.653 million yen in 2018 and 2.794 million yen in 2020.”

Comment 4-2�

The authors' statement that excluded participants were older and in poorer health is helpful, but this is not documented in the manuscript. Given that approximately 40,000 participants were excluded, please add either a supplementary table comparing baseline characteristics of included versus excluded participants, or a brief paragraph in the Methods or Results describing the differences, with an accompanying limitation on potential selection bias.

Response 4-2�

Thank you for this valuable comment. In response, we added Supplementary Table S1 comparing the baseline characteristics of the analytic sample and excluded participants, including individuals with missing data. We also added a corresponding description to the Results section (Lines 367–372) and included an additional statement in the Limitations section (Lines 507–510) regarding the potential for selection bias.

Results (Lines 367–372)

“Table S1 compares the baseline characteristics of the analytic sample and excluded participants, including individuals with missing data. The excluded participants were generally older and less educated than those included in the analytic sample. Specifically, the mean age of excluded participants was 75.3 years, compared with 71.7 years in the analytic sample, and the proportion with ≥13 years of education was lower among excluded participants (17.4%) than among the analytic sample (40.4%).”

Limitations (Lines 517–510)

“In addition, excluded participants were generally older and less educated than those included in the analytic sample. Therefore, selection bias resulting from participant exclusion cannot be ruled out, and the findings may be less generalisable to older adults with poorer socioeconomic profiles. ”

Attachment Submitted filename: Response to Reviewers.docx

# Editor report: Decision Letter 2

**DOI:** 10.1371/journal.pone.0353850.r005

**Authors:** Ryota Sakurai

30 Jun 2026

Associations Between Health-Related Social Capital and Oral Frailty Risk Among Older Adults: A Stratified Analysis by Socioeconomic Status in the JAGES Longitudinal Study

PONE-D-25-65610R2

Dear Dr. Tadaka,

We’re pleased to inform you that your manuscript has been judged scientifically suitable for publication and will be formally accepted for publication once it meets all outstanding technical requirements.

Within one week, you’ll receive an e-mail detailing the required amendments. When these have been addressed, you’ll receive a formal acceptance letter and your manuscript will be scheduled for publication.

An invoice will be generated when your article is formally accepted. Please note, if your institution has a publishing partnership with PLOS and your article meets the relevant criteria, all or part of your publication costs will be covered. Please make sure your user information is up-to-date by logging into Editorial Manager at Editorial Manager® and clicking the ‘Update My Information' link at the top of the page. For questions related to billing, please contact billing support.

If your institution or institutions have a press office, please notify them about your upcoming paper to help maximize its impact. If they’ll be preparing press materials, please inform our press team as soon as possible -- no later than 48 hours after receiving the formal acceptance. Your manuscript will remain under strict press embargo until 2 pm Eastern Time on the date of publication. For more information, please contact onepress@plos.org.

Kind regards,

Ryota Sakurai, Ph.D.

Academic Editor

PLOS One

Additional Editor Comments (optional):

The revisions have satisfactorily addressed the previous concerns, and the manuscript is now suitable for publication. Thank you for your effort.

Reviewers' comments:

# Editor report: Acceptance letter

**DOI:** 10.1371/journal.pone.0353850.r006

**Authors:** Ryota Sakurai

PONE-D-25-65610R2

PLOS One

Dear Dr. Tadaka,

I'm pleased to inform you that your manuscript has been deemed suitable for publication in PLOS One. Congratulations! Your manuscript is now being handed over to our production team.

At this stage, our production department will prepare your paper for publication. This includes ensuring the following:

* All references, tables, and figures are properly cited

* All relevant supporting information is included in the manuscript submission,

* There are no issues that prevent the paper from being properly typeset

You will receive further instructions from the production team, including instructions on how to review your proof when it is ready. Please keep in mind that we are working through a large volume of accepted articles, so please give us a few days to review your paper and let you know the next and final steps.

Lastly, if your institution or institutions have a press office, please let them know about your upcoming paper now to help maximize its impact. If they'll be preparing press materials, please inform our press team within the next 48 hours. Your manuscript will remain under strict press embargo until 2 pm Eastern Time on the date of publication. For more information, please contact onepress@plos.org.

You will receive an invoice from PLOS for your publication fee after your manuscript has reached the completed accept phase. If you receive an email requesting payment before acceptance or for any other service, this may be a phishing scheme. Learn how to identify phishing emails and protect your accounts at https://explore.plos.org/phishing.

If we can help with anything else, please email us at customercare@plos.org.

Thank you for submitting your work to PLOS ONE and supporting open access.

Kind regards,

PLOS ONE Editorial Office Staff

on behalf of

Dr. Ryota Sakurai

Academic Editor

PLOS One
